# Reactive fragments targeting carboxylate residues employing direct to biology, high-throughput chemistry†

**DOI:** 10.1039/d2md00453d

**Published:** 2023-02-22

**Authors:** Ross P. Thomas, Emma K. Grant, Eleanor R. Dickinson, Francesca Zappacosta, Lee J. Edwards, Michael M. Hann, David House, Nicholas C. O. Tomkinson, Jacob T. Bush

**Affiliations:** a GlaxoSmithKline Gunnels Wood Road Stevenage Hertfordshire SG1 2NY UK Jacob.x.Bush@GSK.com; b Department of Pure and Applied Chemistry, University of Strathclyde 295 Cathedral Street Glasgow G1 1XL UK Nicholas.Tomkinson@strath.ac.uk; c GlaxoSmithKline South Collegeville Road Collegeville PA 19426 USA

## Abstract

The screening of covalent or ‘reactive’ fragment libraries against proteins is becoming an integral approach in hit identification, enabling the development of targeted covalent inhibitors and tools. To date, reactive fragment screening has been limited to targeting cysteine residues, thus restricting applicability across the proteome. Carboxylate residues present a unique opportunity to expand the accessible residues due to high proteome occurrence (∼12%). Herein, we present the development of a carboxylate-targeting reactive fragment screening platform utilising 2-aryl-5-carboxytetrazole (ACT) as the photoreactive functionality. The utility of ACT photoreactive fragments (ACT-PhABits) was evaluated by screening a 546-membered library with a small panel of purified proteins. Hits identified for BCL6 and KRAS^G12D^ were characterised by LC-MS/MS studies, revealing the selectivity of the ACT group. Finally, a photosensitised approach to ACT activation was developed, obviating the need for high energy UV-B light.

## Abstract 2

We present a carboxylate-targeting reactive fragment screening platform using 2-aryl-5-carboxytetrazole (ACT) as the photoreactive functionality. This work will provide a simple accessible method to rapidly discover tool molecules to interrogate important biological targets.

## Graphical abstract

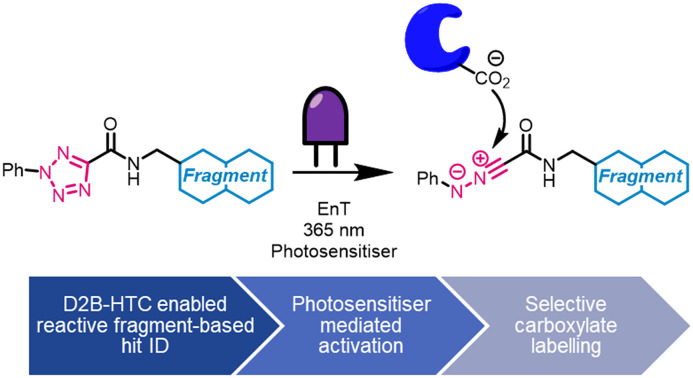

## Introduction

**Fig. 1:**
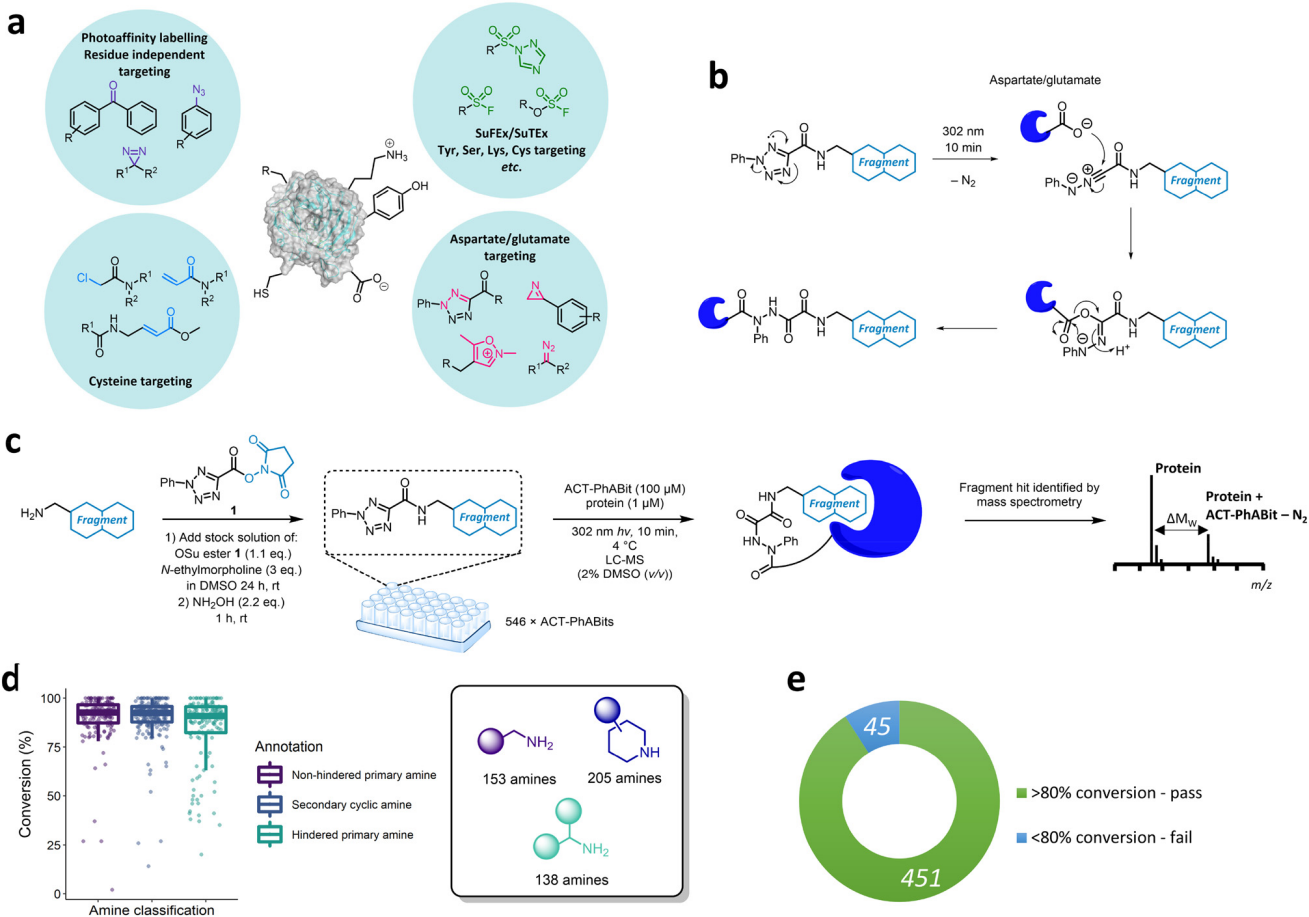
a) Methods for covalent modification of different amino acid side chains. b) Proposed mechanism for the photoactivated covalent capture of carboxylate residues using the 2-aryl-5-carboxytetrazole (ACT) functionality. c) Direct-to-biology high-throughput chemistry (D2B-HTC) synthesis of a 546-membered ACT-PhABit library in 384-well plates and direct screening of crude reaction products without purification with a protein target. d) Boxplot for the conversion of the ACT-PhABit reactions grouped by the classification of the amine structure. Amines were classified according to the following categories: hindered primary amines, unhindered primary amines and secondary cyclic amines. 50 reactions were omitted on account of impure amine starting materials or an inability to calculate conversion. e) Overall success rate of the ACT-PhABit synthetic protocol.

In recent years, small molecules with a covalent mechanism of action have emerged as powerful modalities in medicinal chemistry and chemical biology.^1–6^ A key benefit of covalent inhibition is the ability to target shallow binding pockets and protein–protein interaction (PPI) interfaces through enhanced potency and prolonged target occupancy.^7,8^ Recently, there has been a growing interest in the application of reactive fragments for the development of targeted covalent inhibitors (TCIs).^9–12^ Reactive fragments combine the efficient coverage of chemical space offered by fragments with a reactive moiety to enable robust covalent capture of weak fragment-protein binding interactions.^13–16^ Early reactive fragment screening focussed on targeting cysteine residues (Fig. 1a).^4,10,12,13,17^ Notably, cysteine reactive fragments were employed for targeting the challenging oncology target KRAS^G12C^, culminating with the development of sotorasib (AMG510).^1^ The low frequency of cysteine (∼2%)^18,19^ in the proteome has prompted research into alternative covalent modalities that can selectively target less nucleophilic amino acids including tyrosine and lysine (Fig. 1a).^20–28^

To date, there has been limited research on selectively targeting aspartate (Asp) and glutamate (Glu) residues (Fig. 1a).^29–33^ The high proteome occurrence of Asp and Glu residues (>12% combined) combined with the propensity for these polar residues to be located on the surface of proteins suggests they could offer a useful handle for the development of covalent ligands.^18,19,34^ Recently a number of carboxylate targeting groups have been employed in the development of covalent ligands, including diazo modalities,^33,35,36^ isoxazolium derivatives^30^ and 2*H*-azirines (Fig. 1a).^29^ Tetrazole-moieties offer a photoactivable approach towards irreversible carboxylate modification.^31,34,37^ Diarylsubstituted tetrazoles were reported by Bach *et al.* to enable proteome-wide profiling of aspartate and glutamate residues in bacteria.^31^ Similarly, 2-aryl-5-carboxytetrazoles (ACTs) represent a photoactivatable carboxylate-targeting covalent functionality that features a carboxylic acid handle for facile derivatisation with suitable recognition elements (Fig. 1a and b).^32,38^ Photolysis using ultraviolet (UV) light (302 nm) affords the nitrilimine reactive species, which can react with a carboxylate residue *via* an *O* → *N* acyl shift to furnish the corresponding 1,2-diacylhydrazine (Fig. 1b).^31,32^ The ACT-derived nitrilimine intermediate exhibits a longer lifetime than common photoactivable diazirines (>500 μs *cf.* carbene <2 ns), which can lead to enhanced crosslinking yields.^32,39–41^

While a photoreactive mechanism does not offer the boost in potency observed with electrophilic covalent mechanisms, it can be advantageous in enabling the discovery of ligands with good non-covalent affinity. Previously we have reported the screening of diazirine containing fragments for the discovery of ligands for proteins of interest.^14,42^ A challenge with this approach can be the low crosslinking yields achieved with carbenes, and it was anticipated that the nitrilimine furnished by the photoactivation of the ACT reactive group may offer improved crosslinking yields.

Herein, we describe the development of an ACT reactive fragment platform that allows the capture of fragment–protein interactions *via* selective carboxylate side chain modification. A library of ACT-derived fragments was synthesised using a direct-to-biology, high-throughput chemistry approach and screened against a panel of therapeutically relevant proteins including BRD4-BD1, BCL6, carbonic anhydrase I & II (CAI/CAII) and KRAS^G12D^. Intact-protein mass spectrometry enabled identification of hits which included established chemotypes as well as novel binding motifs for these therapeutically relevant proteins. The hits were further characterised to determine the site of modification, revealing a potentially novel binding site on KRAS. Finally, an energy transfer (EnT) approach towards activation of the ACT moiety was also developed, allowing the use of longer wavelength light that is less damaging to the protein.

## Results and discussion

### Synthesis of an ACT-PhABit library

We anticipated that a direct-to-biology high-throughput chemistry (D2B-HTC) platform would enable access to a library of ACT-derived reactive fragments for expedient evaluation as a carboxylate-targeting screening strategy (Fig. 1c).^42^ This methodology employs 384-well plate-based amide couplings of amine fragments with an activated succinimide ester bearing the reactive functionality (1, Fig. 1c). The crude reaction products were screened without purification with the protein target in a ‘direct-to-biology’ approach.

To generate the ACT-PhABit library, we selected 546 amine fragments that afforded high conversion to the corresponding amides in a HTC-PhABit library.^42^ These included primary unhindered-, primary hindered- and secondary cyclic-alkylamines (ESI† section 3.1) (for library properties see Fig. S1†).^42^ Amines were plated as singletons in 384-well plates (10 mM DMSO) followed by addition of a DMSO solution of OSu ester 1 and *N*-ethylmorpholine (NEM). The plates were sealed and incubated at room temperature for 24 h to afford the crude ACT-PhABits. The reactions were quenched with NH_2_OH_(aq)_ (2.2 eq., 1 h, rt) to remove unreacted OSu ester 1, preventing potential non-specific labelling of nucleophilic amino acid residues on the protein surface.^42^ LC-MS analysis of the plates indicated a high success rate of 91% for the 496 reactions quantified (Fig. 1d and e, for full breakdown of reaction qualification see Fig. S2†). Of the remaining 50, conversion could not successfully be calculated or the amine starting material was impure.

### Screening the ACT-PhABit library

Following the generation of the D2B-ready ACT-PhABits, a panel of proteins, BCL6, BRD4-BD1, CAI & CAII, KRAS^G12D^ and myoglobin, were selected for screening. BRD4, CAI and CAII were selected since they were known to bind to chemotypes present in the library which could serve as positive controls. The KRAS^G12D^ mutant is a common mutation in cancers and was therefore selected to investigate whether Asp12 could be specifically modified by the ACT-derived nitrilimine reactive species.^43^ BCL6 is a transcription factor that is often dysregulated in B-cell lymphomas and autoimmune diseases.^22,44^ A number of inhibitors have been reported that bind in the shallow groove formed at the BCL6 homodimeric interface, disrupting co-repressor PPIs and restoring BCL6 target genes.^22,44–47^ BCL6 therefore served as a target to investigate whether the ACT-fragment could ligate a shallow PPI pocket.

**Fig. 2:**
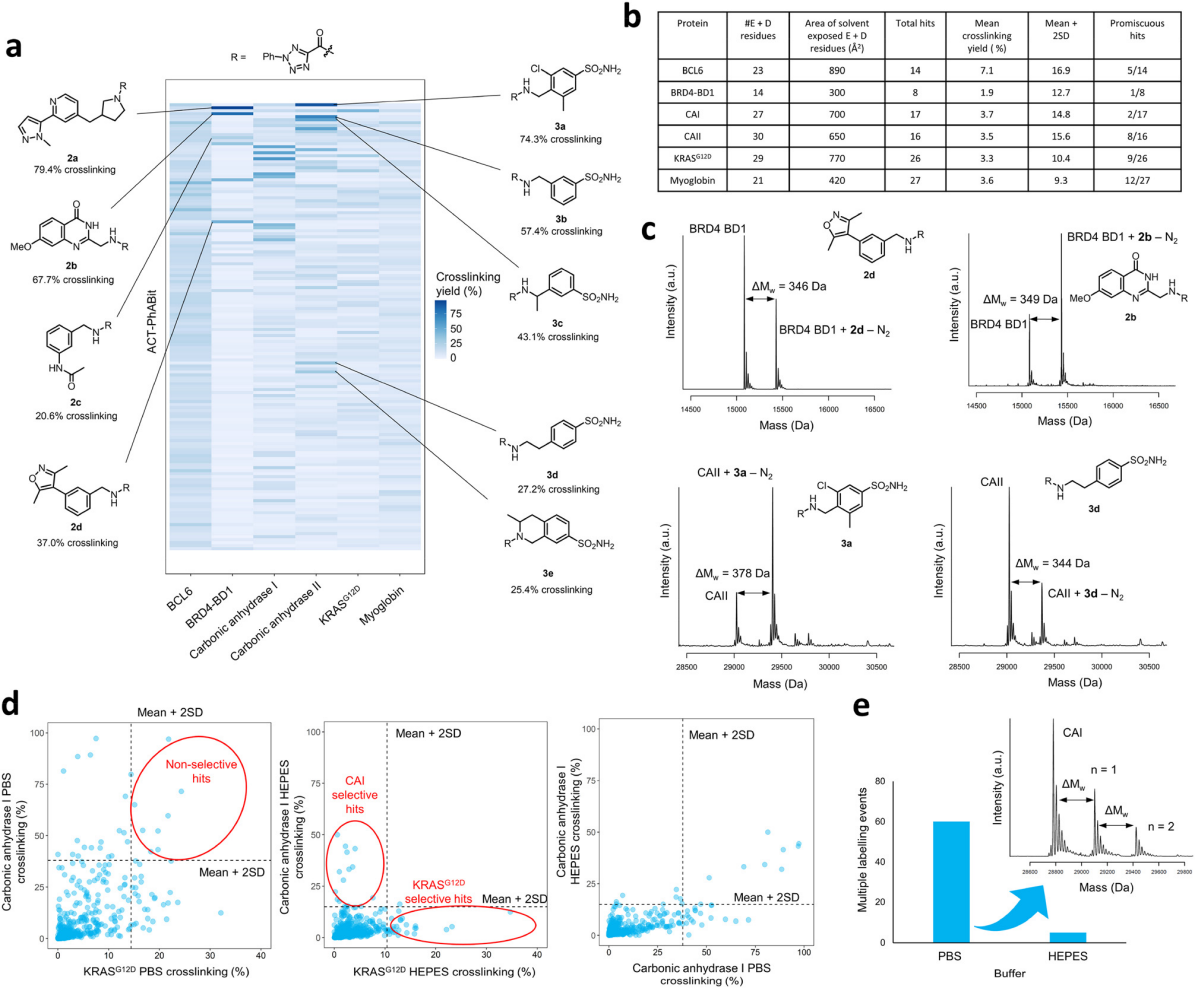
a) The ACT-PhABit library (100 μM) was irradiated (302 nm, 10 min) with protein (1 μM) in HEPES buffer, before analysis by intact-protein LC-MS. Heatmap displaying the crosslinking yields of the top 150 ACT-PhABits (ordered top to bottom by average crosslinking across the six proteins) with a panel of proteins, highlighting hit structures from the BRD4-BD1 and carbonic anhydrase II (CAII) screens. b) Screening summary data for the panel of proteins that were screened against the 546-membered ACT-PhABit library, including the total number of carboxylate residues (E = glutamate, D = aspartate) in each protein and area of solvent exposed carboxylate residues. c) Structures and corresponding mass spectra of selected BRD4-BD1 and CAII hits. d) Comparison of crosslinking yields across the 546-membered ACT-PhABit library (100 μM) with carbonic anhydrase I (CAI) and KRAS^G12D^ (each 1 μM) in two different buffers, PBS and HEPES. Hits are identified as those displaying crosslinking yields >(mean + 2 × standard deviation), indicated by the dashed lines. e) Number of multiple labelling events across the ACT-PhABit screen against CAI in both buffers, PBS and HEPES, alongside an exemplar mass spectrum displaying multiple labelling.

The proteins were incubated with the library (100 μM ACT-PhABit, 1 μM protein) and irradiated (10 min, 302 nm). Intact-protein LC-MS was used to identify any light-induced covalent crosslinking events by detection of a mass shift corresponding to [protein + ACT-PhABit − N_2_]. Hits were characterised as ACT-PhABits that modified the protein with a crosslinking yield >mean + 2 × standard deviation (mean + 2SD). The mean crosslinking that was observed across the panel of proteins ranged from 1.9% (BRD4-BD1) to 7.1% (BCL6) (Fig. 2a and b and S3†), indicating a low extent of background, non-specific labelling. A heatmap of crosslinking in all screens highlighted selective crosslinking between specific fragments for each protein, supporting that crosslinking is driven by recognition mediated by the fragment moiety (Fig. 2a). Validation that the platform could identify true hits was provided by the identification of known binders, such as acetyl lysine mimetics (*e.g.* dimethylisoxazole) for BRD4-BD1 (Fig. 2a and c, 2a–d)^48–50^ and a number of primary sulfonamide hits (Fig. 2a and c, 3a–e) for CAII, which are known to bind in the Zn^2+^ binding site.^51^ The screen identified 14 ACT-PhABit hits for BCL6, 9 of which were not hits for any other protein, whilst 26 were identified for KRAS^G12D^, 17 of which were selective (Fig. 2b and S4 and S5†).

### Specificity of crosslinking and buffer selection

A key consideration in screening of reactive fragments is deconvolution of specific and non-specific binding events.^52^ Typically, reactive groups and screening conditions are tailored to maximise crosslinking of true binders, whilst minimising non-specific crosslinking.^53–56^ In the case of ACT-PhABits, the nitrilimine intermediate has a reported lifetime of >500 μs,^41^ which is a relatively long lifetime (*cf.* carbene <2 ns).^39^ It was therefore anticipated that the buffer could have a significant effect on the quenching of the reactive species, and thus the extent of non-specific crosslinking. The screens with CAI and KRAS^G12D^ were repeated in two buffers, phosphate buffered saline (PBS) (pH 7.2) and HEPES (50 mM, pH 7.4). The mean crosslinking yield for CAI was 8.4% in PBS and 3.7% in HEPES, and for KRAS^G12D^ was 4.0% in PBS and 3.3% in HEPES respectively. These results suggest that screening in HEPES reduces the formation of non-specific crosslinking with carboxylate residues, likely through nucleophilic quenching of the nitrilimine species by the buffer (Fig. S6†). The occurrence of non-specific crosslinking in PBS was further confirmed by the observation that several fragments crosslinked to both CAI and KRAS^G12D^ in PBS (Fig. 2d, left plot), whilst HEPES afforded selective hits for each protein (Fig. 2d, middle plot). Analysis of the CAI data in the two buffers highlighted that many additional crosslinkers were observed in PBS (Fig. 2d, right plot). Finally, the number of multiple labelling events observed was calculated to provide an indicator of non-specific crosslinking. A total of 60 multiple labelling events were identified across the CAI PBS buffer screen compared to 5 across the CAI HEPES buffer screen (Fig. 2e). The attenuation of crosslinking in HEPES was thus anticipated to limit the occurrence of false positive hits caused by non-specific crosslinking.

With optimised screening conditions in place, it was observed that crosslinking yields of hits from the ACT-PhABit screen were notably improved over the previous reported screens using a diazirine library. The screen with CAII afforded 16 ACT-PhABit hits with over 10% crosslinking (*cf.* just 2 with a diazirine library) and with KRAS^G12D^ 26 ACT-PhABit hits yielded over 10% crosslinking (*cf.* just 6 with the diazirine library).^14^

### Follow-up studies on KRAS^G12D^ and BCL6 hits

**Fig. 3:**
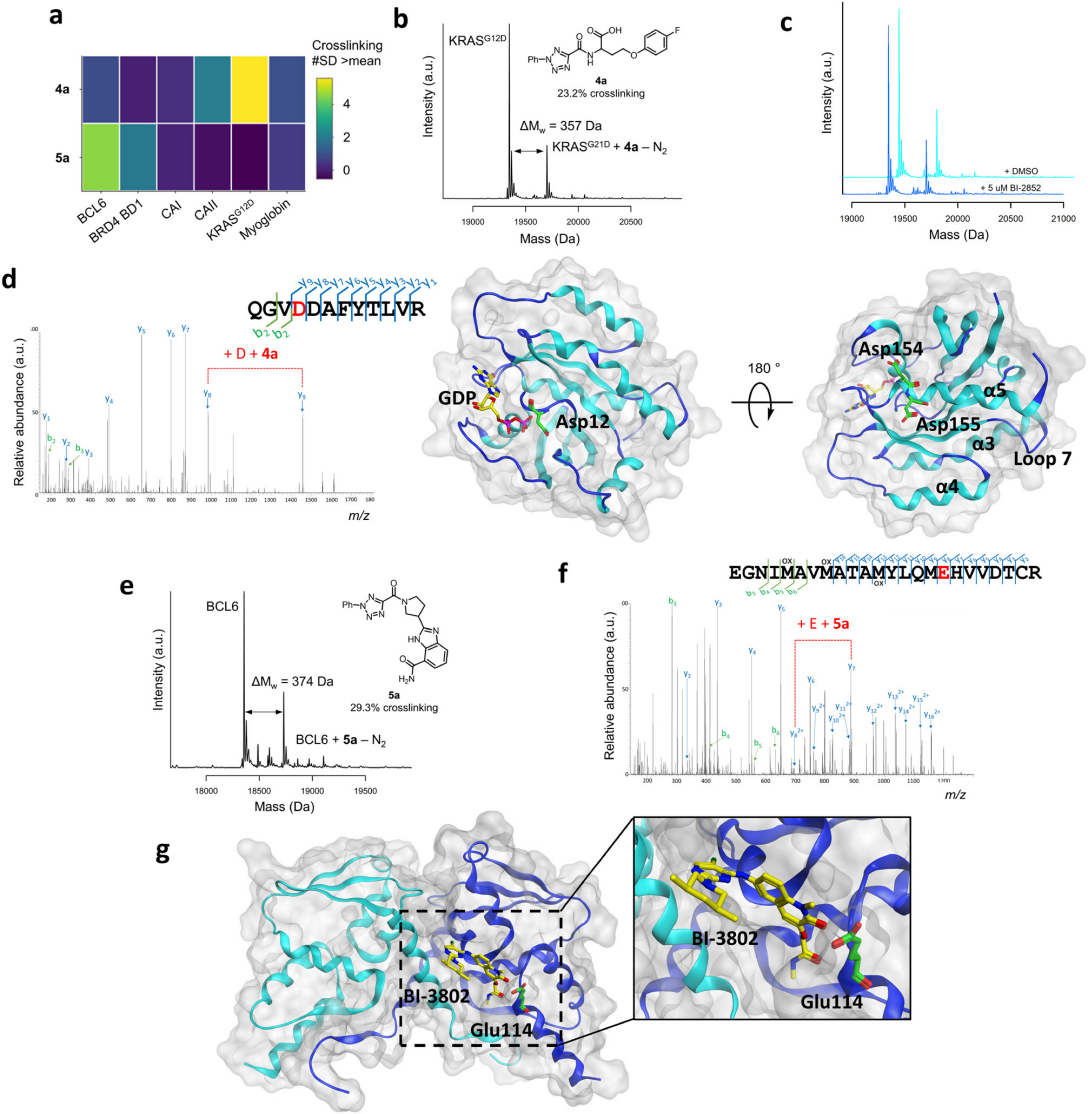
a) Crosslinking yields (#SD >mean) of compounds 4a and 5a across the panel of proteins that were screened, shown by a heatmap. b) Structure and corresponding mass spectrum of ACT-PhABit 4a (100 μM) as the crude reaction product crosslinked to KRAS^G12D^ (1 μM). c) Crosslinking competition study between purified ACT-PhABit 4a (50 μM) and known KRAS^G12D^ inhibitor, BI-2852 (5 μM). d) MS/MS spectrum of the peptide _151_QGVDDAFYTLVR_162_ crosslinked to 4a, indicating Asp154 as the site of crosslinking and the corresponding X-ray crystal structure of GDP-bound (yellow) KRAS^G12D^ (PDB: 5US4) highlighting Asp154 (green) as the major site of crosslinking. e) Structure and corresponding mass spectrum of ACT-PhABit 5a (100 μM) as the crude reaction product crosslinked to BCL6 (1 μM). f) MS/MS spectrum of the peptide _98_EGNIMAVMATAMYLQMEHVVDTCR_121_ crosslinked to 5a, indicating Glu114 as the site of crosslinking. g) The corresponding X-ray crystal structure of BCL6 (PDB: 5MW2) highlighting Glu114 (green) as the major site of crosslinking and known binder BI-3802 (yellow).

Selected hits from the KRAS^G12D^ and BCL6 screens were chosen for further analysis (Fig. 3a). Compound 4a was the KRAS^G12D^ hit with the highest crosslinking yield (23.2%, Fig. 3b) and displayed good selectivity across the six proteins screened (Fig. 3a). Following resynthesis and purification, compound 4a displayed enhanced crosslinking of 42.2%, likely due to the lower concentration of the HTC compound in the crude reaction mixture. Competition studies between compound 4a (50 μM) with known switch I/II KRAS^G12D^ inhibitor BI-2852 (5 μM), a nanomolar inhibitor,^57^ indicated that these compounds did not compete for the same site, suggesting an alternative binding site for compound 4a (Fig. 3c). To identify the site of binding, 4a-labelled KRAS^G12D^ was digested trypsin/LysC and analysed by LC-MS/MS, which identified peptide _151_QGVD*DAFYTLVR_162_ as carrying the modification (357.1125 Da) on Asp154 (Fig. 3d). A minor amount of modification also observed on Asp155. Residues Asp154/Asp155 are located away from the functional GDP switch I/II binding site, consistent with the competition data with BI-2852. These residues are located near the allosteric p3 KRAS site, which is formed from residues in loop 7 and the C-terminal end of the α5 helix, and therefore may present an opportunity for optimisation of a novel covalent ligand for this protein (Fig. 3d).^58,59^

The BCL6 hit 5a (Fig. 3a and e) was also resynthesised, purified and binding was confirmed by intact-protein LC-MS (31.7% *versus* 29.3% crosslinking for purified *versus* HTC, respectively). LC-MS/MS analysis identified peptide _98_EGNIMAVMATAMYLQME*HVVDTCR_121_ as carrying the modification (374.1491 Da) on Glu114 (Fig. 3f). Glu114 is located at one end of the lateral grooved formed at the BCL6 homodimeric interface (Fig. 3g).^22^ Multiple reported inhibitors also engage with BCL6 in the lateral groove (*e.g.* BI-3802,^47^Fig. 3g), indicating that ACT-PhABit 5a is binding and crosslinking in a functional site, supporting that the covalent modification is driven by a molecular recognition event.^22^

These data indicate that under suitable screening conditions it is possible to capture specific fragment–protein interactions using the ACT functionality, even for challenging protein pockets, offering an opportunity for the development of specific chemical binders for therapeutically relevant proteins. The hit compounds for KRAS and BCL6 are anticipated to have, at best, weak biochemical activity and are not be expect to have sufficient potency or selectivity to show activity in cells, however, they may represent effective starting points for further development toward more potent and selective tools.

### Developing a photosensitiser-based approach for ACT-based photoaffinity labelling

**Fig. 4:**
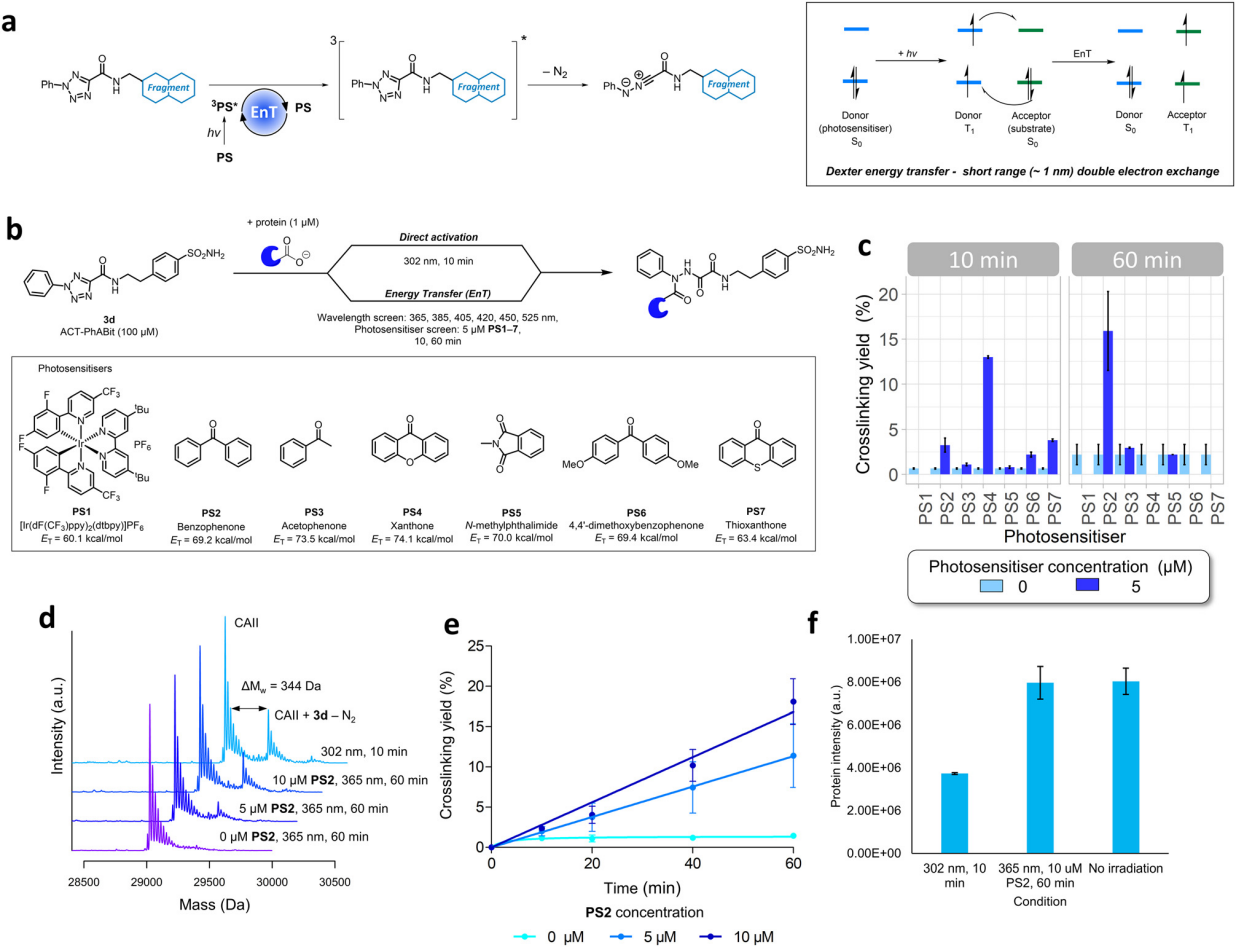
a) Alternative pathway of ACT activation *via* Dexter energy transfer (EnT) mechanism. An EnT step from a suitably excited photosensitiser was anticipated to indirectly photolyse the ACT functionality and form the respective nitrilimine reactive species. b) Proposed EnT screening strategy to identify suitable photosensitisers and associated wavelengths of activation compared to traditional high energy UV light (302 nm) direct activation. Wavelengths and photosensitisers that were screened are shown. c) Crosslinking yields observed when ACT-PhABit 3d (100 μM) was irradiated at 365 nm (10 & 60 min) with CAII (1 μM) in the presence of each of the seven photosensitisers (**PS1**–**7**, 5 μM). Some data has been omitted on account of protein damage, confounding analysis. d) Comparison of crosslinking yields between direct activation (302 nm, 10 min) and energy transfer pathways (0, 5, 10 μM **PS2**, 365 nm, 60 min) of ACT-PhABit 3d (100 μM) with CAII (1 μM). e) Time and photosensitiser concentration dependent crosslinking of ACT-PhABit 3d (100 μM) with CAII (1 μM) upon activation with 365 nm light. f) Comparison of protein intensity by mass spectrometry after irradiation with 302 nm (10 min), 365 nm (60 min) + **PS2** (10 μM) and no irradiation.

The high energy UV-B light required for the activation of ACT-PhABits (302 nm) is highly damaging to proteins, which absorb light <320 nm.^60,61^ Amongst the most chromophoric amino acids are tryptophan, tyrosine, histidine, cysteine and phenylalanine, which absorb UV-B light (280–320 nm), leading to radical-based side reactions and scission pathways.^60–62^ MacMillan and co-workers recently reported that blue light (450 nm) in combination with a suitable photosensitiser could be used for the activation of diazirines *via* Dexter energy transfer (EnT).^63^ It was therefore postulated that the ACT functionality could be activated in a similar fashion *via* a Dexter energy transfer (EnT) mechanism using a combination of longer wavelength, lower energy light (*hv*) and a suitable photosensitiser (PS).^32,63–65^ Energy transfer is a photophysical process whereby a photosensitiser (donor), upon excitation and intersystem crossing (ISC) to a long lived (>100 ns) triplet excited state (T_1_), is able to sensitise a substrate (acceptor) to its corresponding excited state (T_1_) *via* a double electron exchange (Fig. 4a).^64,65^ Such an EnT approach, which would utilise longer wavelength light, was anticipated to offer more tolerant and less damaging irradiation conditions towards larger, more chromophoric, proteins, whilst also being more tolerant of photo-sensitive functional groups in the ACT-PhABits.^66,67^

A selection of seven photosensitisers were chosen for investigation based on their high triplet energies (*E*_T_) (**PS1**–**7**, Table S1†).^64,65,68^ CAII (1 μM) was incubated with ACT-PhABit 3d (100 μM) before irradiation at six wavelengths (365–525 nm, 10–60 min) in the presence of each of the seven photosensitisers (5 μM) (Fig. 4b). Crosslinking events were observed in the cases of benzophenone (**PS2**) and xanthone (**PS4**) using 365 nm light (Fig. 4c and S8 and S9†), affording 15.9% and 13.0% crosslinking (*n* = 2), respectively. No crosslinking was observed using 365 nm light in the absence of the photosensitisers (Fig. 4c and S8 and S9†). For comparison, ACT-PhABit 3d was also irradiated at 302 nm for 10 min as a control, affording a crosslinking yield of 23.9% (Fig. 4d). Benzophenone **PS2** was selected for further investigation due to the cleaner mass spectra observed when compared to xanthone **PS4** (Fig. S8 and S9†). Additional studies utilising **PS2** as an ACT photosensitiser revealed the time- and concentration-dependent behaviour of the crosslinking (Fig. 4d and e and S10†). A maximum crosslinking yield of 18.1% was obtained by irradiating ACT-PhABit 3d in the presence of 10 μM **PS2** for 60 min.

Finally, the effect of irradiation on protein integrity was investigated. The EnT irradiation process at 365 nm (60 min) + **PS2** (10 μM) resulted in negligible reduction of CAII protein MS signal whereas irradiation at 302 nm (10 min) showed a significant decrease in the protein MS signal (Fig. 4f). Furthermore, the EnT conditions were comparable to a control sample of CAII that was subjected to no irradiation. Thus, this EnT approach offers a milder method for the activation of ACT groups for photolabeling compared to the original direct activation conditions, whilst attaining comparable crosslinking yields.

## Conclusion

Reactive fragment screening has become integral in hit identification in recent years. While the focus of these efforts has centred on targeting cysteine residues, modalities that enable covalent modification ‘beyond cysteine’ are highly sought after in order to expand the ligandable proteome. The high proteome occurrence of carboxylate-containing residues, aspartate and glutamate (>12%), makes them good targets for reactive fragment screening. This work describes the development of a direct-to-biology photoreactive carboxylate-targeting reactive fragment screening platform, which enables the light-induced covalent capture and identification of fragment–protein binding interactions.

The implementation of direct-to-biology high-throughput chemistry (D2B-HTC) allowed the rapid synthesis of an ACT photoreactive fragment (ACT-PhABit) library for direct screening against a panel of therapeutically relevant proteins, which enabled the identification of multiple fragment hits.^42^ The identification of numerous chemotypes known to bind to both BRD4-BD1 and carbonic anhydrase II indicated that specific reversible recognition events were being captured. Further characterisation of BCL6 and KRAS^G12D^ hits by tandem LC-MS/MS revealed the selectivity of the ACT functionality for carboxylate residues and identified both orthosteric and allosteric sites of binding.

Hits afforded higher crosslinking yields than observed previously with diazirine-based fragment screening (PhABits), which facilitates both hit calling and follow-up studies.^14,42^ This is consistent with the formation of a longer-lived nitrilimine intermediate upon photoactivation of the ACT, which reacts preferentially with carboxylate protein side chains. By contrast, the diazirine forms the shorter-lived carbene upon photoactivation, which can be rapidly quenched by water, often leading to low crosslinking yields.

Finally, a novel photosensitised approach towards ACT activation was developed utilising 365 nm light and benzophenone, which obviated the need for damaging UV-B light (302 nm). This modification to the screening platform will be especially valuable with larger/low-stability proteins, which are typically more sensitive to exposure to UV light, often prohibiting the use of photoreactive screening platforms.

The ACT-PhABit screening platform offers an approach towards the development of carboxylate-targeting chemical probes, which presents a novel and complementary addition to the reactive fragment toolbox. Future opportunities will likely lie in the use of electrophilic carboxylate-targeting reactive fragments, such as reactive fragments bearing 2*H*-azirines or isoxazolium salts. The electrophilic nature of these groups could offer improved crosslinking yields by driving towards complete target occupancy over time and could also prove more versatile in biomedical research by obviating the requirement for high energy light.

## Conflicts of interest

There are no conflicts to declare.

## Supplementary Material

MD-014-D2MD00453D-s001
