# Supplementary material for: Reactive fragments targeting carboxylate residues employing direct to biology, high-throughput chemistry
Source: RSC Med Chem. 2023 Feb 22;14(4):671–9. doi: 10.1039/d2md00453d (PMC10131605; doi:10.1039/d2md00453d)
Supplement: MD-014-D2MD00453D-s001 [file MD-014-D2MD00453D-s001.pdf]

Electronic Supplementary Material (ESI) for RSC Medicinal Chemistry.
This journal is © The Royal Society of Chemistry 2023

Reactive fragments targeting carboxylate residues employing direct to biology, high-throughput chemistry

Ross P. Thomas, Emma K. Grant, Eleanor R. Dickinson, Francesca Zappacosta, Lee J. Edwards, Michael M. Hann David House, Nicholas C. O. Tomkinson* and Jacob T. Bush*

Supplementary information

Contents

1 . Supplementary Figures 4

2. General Methods 15

    2.1 Solvents, reagents and consumables 15

    2.2 Irradiation 16

    2.3 Nuclear magnetic resonance (NMR) spectroscopy 17

    2.4 Liquid chromatography-mass spectrometry (LC-MS) for small molecules 17

    2.5 In-plate LC-MS of ACT-PhABit library 18

    2.6 Mass directed automated preparative HPLC (MDAP) 18

    2.7 Infrared Spectroscopy 19

    2.8 Melting Point 19

    2.9 High-resolution mass spectrometry (HRMS) 19

    2.10 TLC and Flash column chromatography 20

    2.11 Intact-protein LC-MS 21

    2.12 Centrifuge 22

    2.13 Protein MOE calculations 22

3. Library synthesis 23

    3.1 Amine selection 23

    3.2 ACT-PhABit synthesis 24

4. Screening the ACT-PhABit library 24

    4.1 ACT-PhABit plate preparation 24

    4.2 Protein addition and irradiation protocol 24

    4.3 Screening the 546-membered ACT-PhABit library 25

5. Follow-up studies: photolysis, competition and LC-MS/MS 26

    5.1 ACT-photolysis studies 26

    5.2 $KRAS^{G12D}$ competition studies with BI-2852 26

    5.3 Site of crosslinking LC-MS/MS studies 27

6. ACT photosensitiser studies 29

    6.1 Photosensitiser screen 29

    6.2 Benzophenone photosensitiser follow-up timecourse 29

    6.3 Protein intensity studies 30

7. Compounds 31

    7.1 Synthesis of ACT-OSu ester **1** 31

    7.2 General procedure for the re-synthesis of ACT-PhABit hits 32

7.3 ACT-PhABit hits ... 32
7.4 Synthesis of $KRAS^{G12D}$ hit **4a** ... 34
8. References ... 38

1 . Supplementary Figures

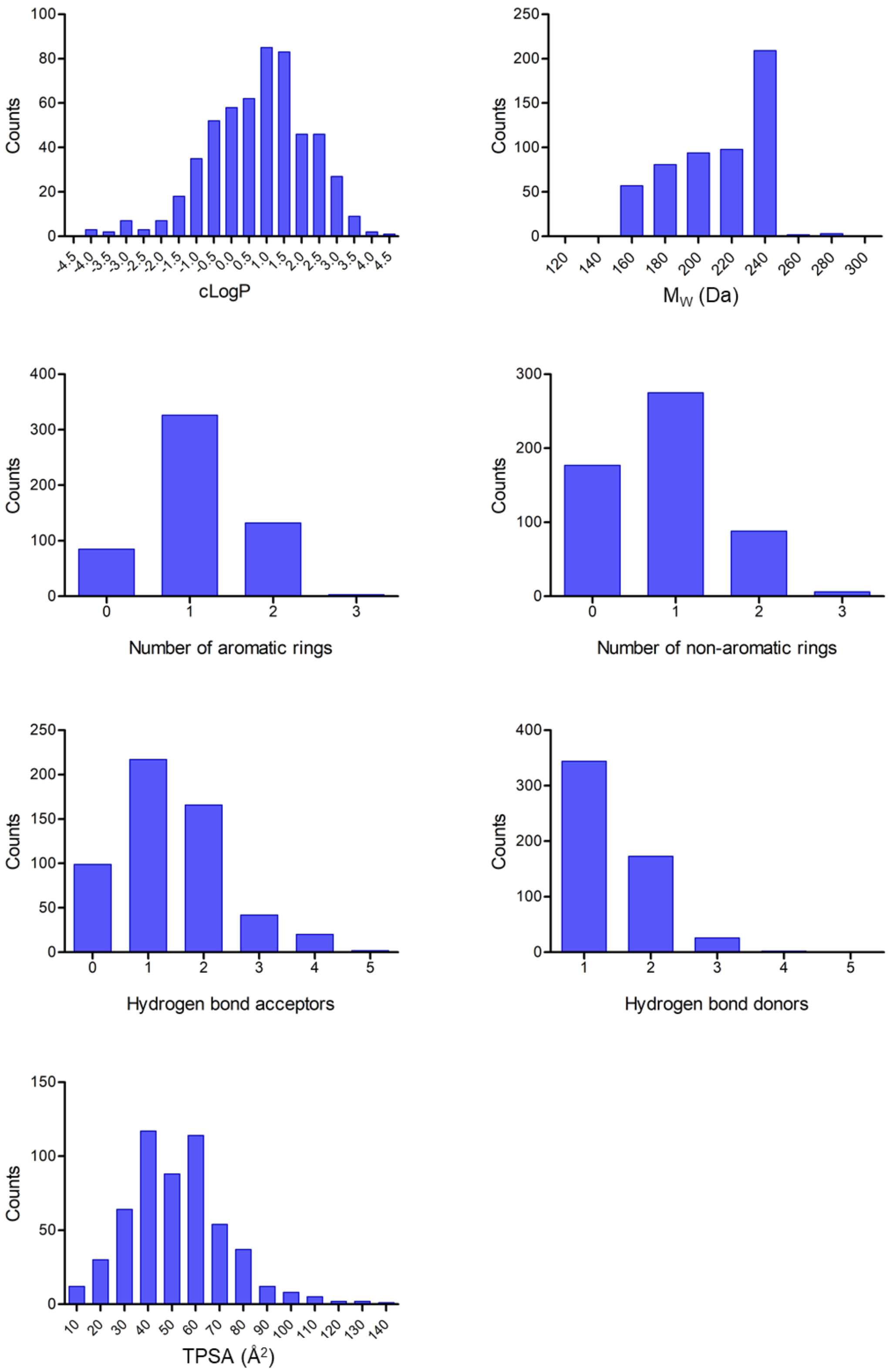

Figure S1. Binned properties of the 546-membered amine fragment library. Free 'uncapped' amines were subjected to the analysis.

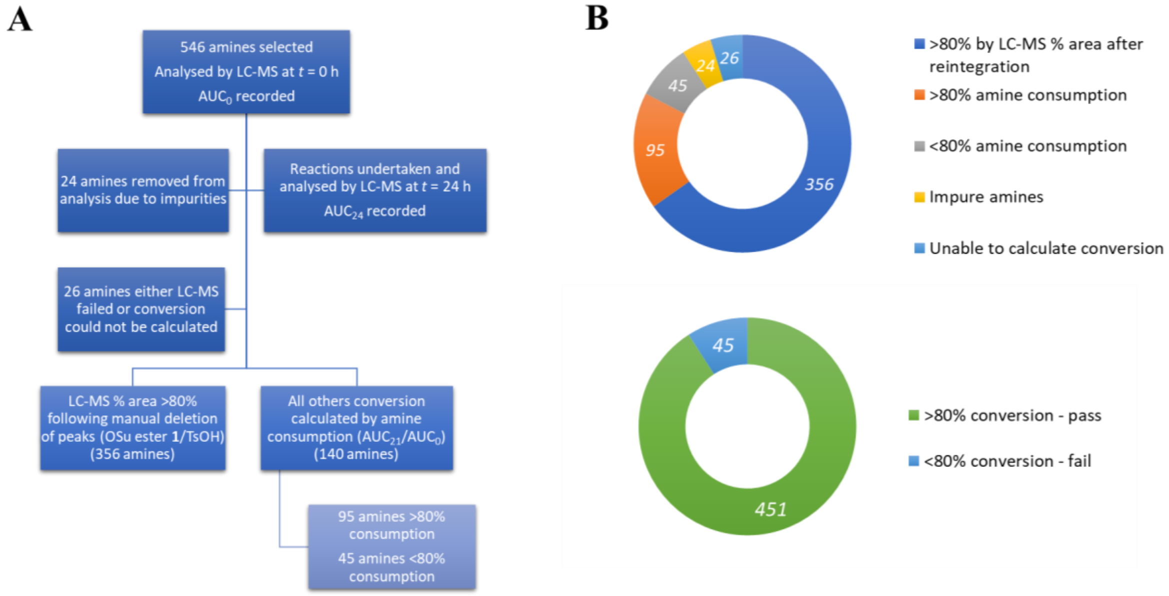

Figure S2a) Flow chart detailing the processing of LC-MS data from the 546 × HTC reactions. Success was determined by the LC-MS % area of the product peak following removal of unreacted ACT OSu ester ***1*** *and TsOH peaks. Where this failed conversion was calculated by consumption of amine using the area under curve (AUC) at the two time points (0 and 24 h). b) Pie charts indicating the rate of success of the HTC protocol.*

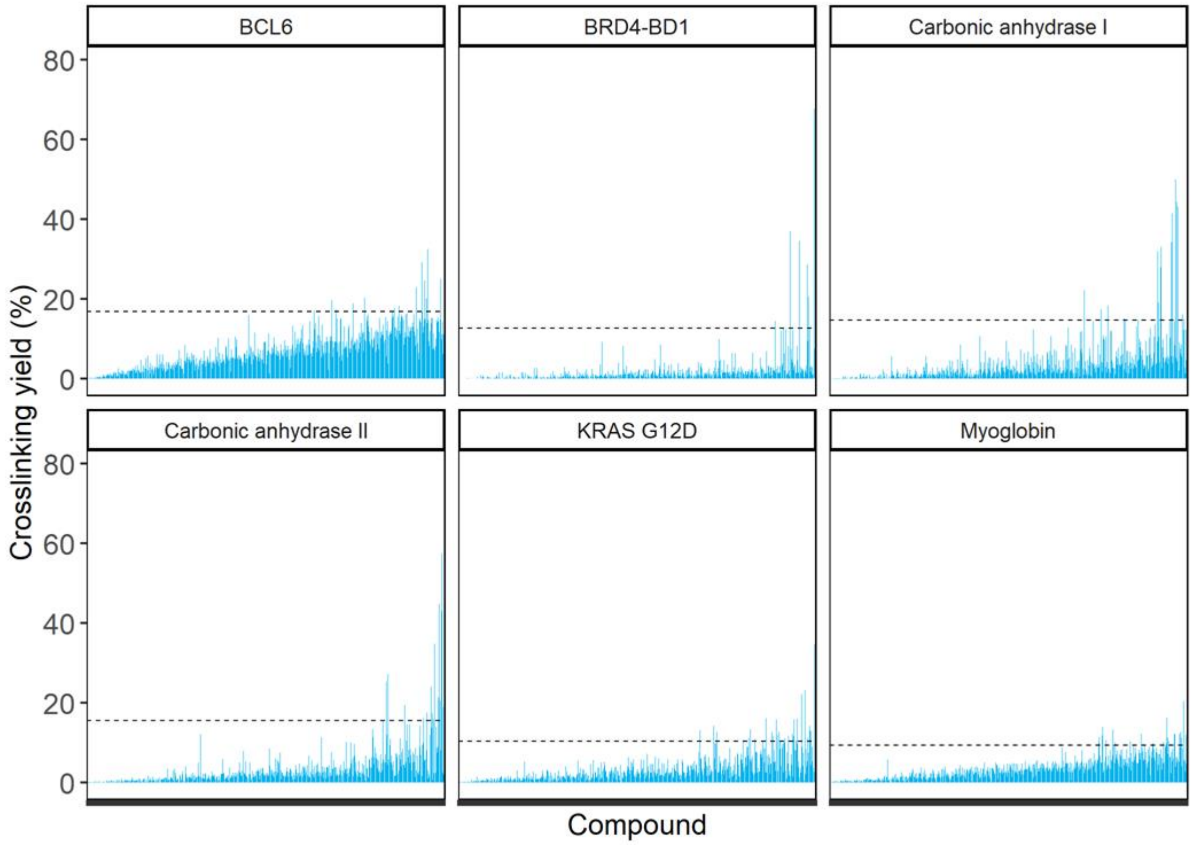

Figure S3. Summary of crosslinking yields of the whole 546-membered ACT-PhABit library against each of the six proteins screened. Compounds were ranked (from right to left) by average crosslinking yield across all six proteins. Dashed lines indicate the mean + 2SD for each screen.

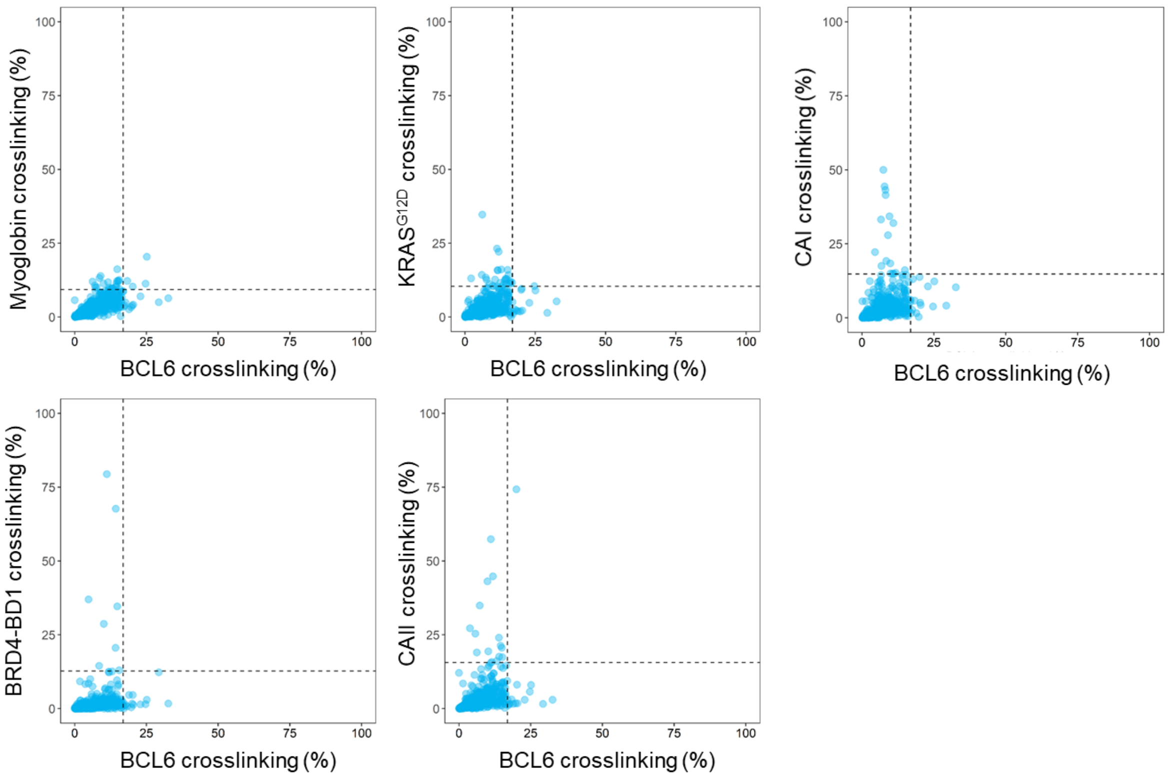

Figure S4. Crosslinking yields of the 546-membered ACT-PhABit library (100 µM) with BCL6 (1 µM) in HEPES compared to the panel of five proteins (1 µM) also screened (CAI, CAII, BRD4-BD1, KRAS^{G12D}, Myoglobin). Dashed lines represent hit cut-off at the mean + 2SD.

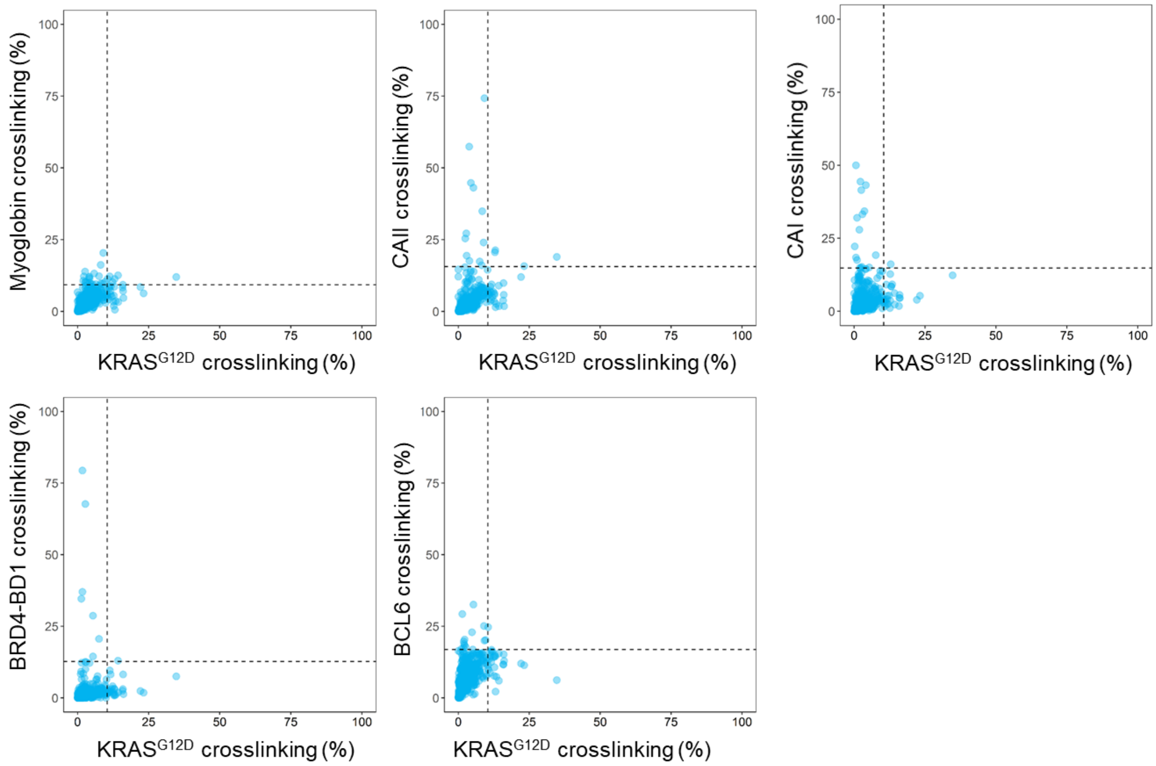

Figure S5. Crosslinking yields of the 546-membered ACT-PhABit library (100 µM) with KRAS^{G12D} (1 µM) in HEPES compared to the panel of five proteins (1 µM) also screened (CAI, CAII, BRD4-BD1, BCL6, Myoglobin). Dashed lines represent hit cut-off at the mean + 2SD.

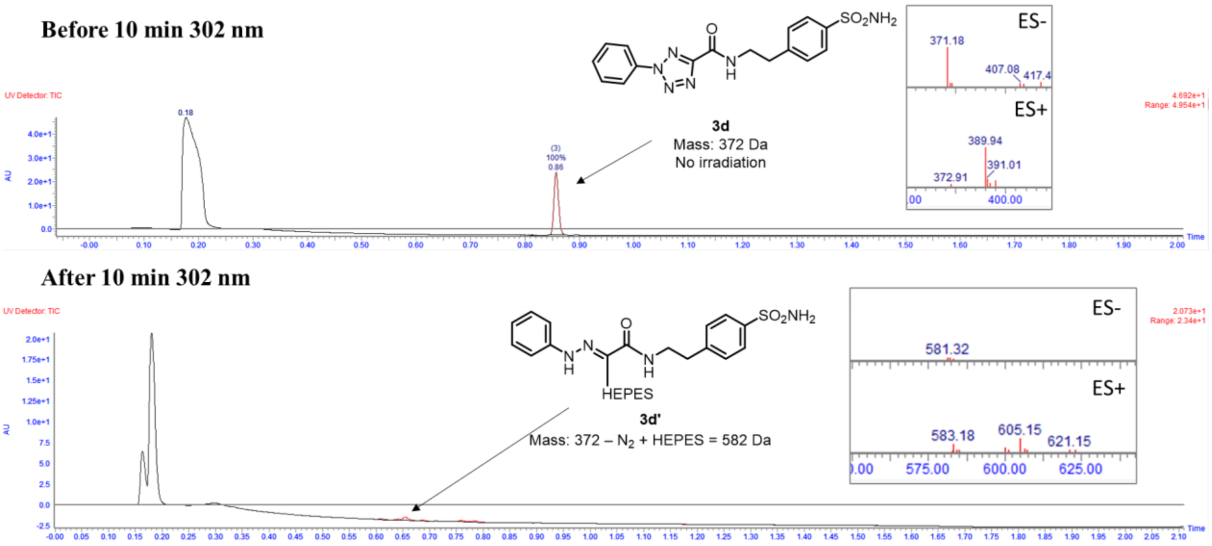

*Figure S6. LC-MS chromatogram of ACT-PhABit **3d** prior to irradiation (top). LC-MS chromatogram of ACT-PhABit **3d** (100 µM) following irradiation (10 min, 302 nm) in 50 mM HEPES buffer (bottom). The major product featured a mass corresponding to [**3d** – N_2 + HEPES], confirming HEPES adduct formation.*

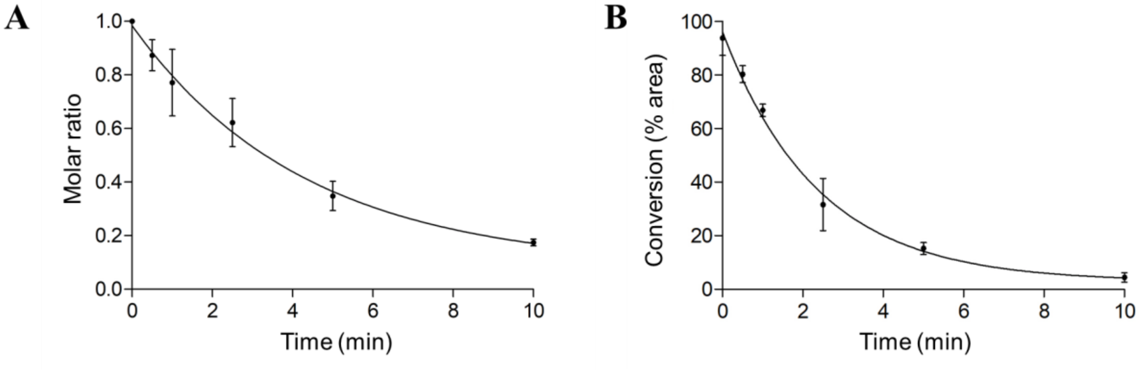

*Figure S7a) Rate of photolysis of ACT-PhABit **3a** in DMSO-d6:D_2O (7:3). Molar ratio after varying lengths of irradiation (302 nm) was calculated by ^{1}H NMR relative to internal standard, dimethylsulfone. Data recorded in duplicate. b) Rate of photolysis of ACT-PhABit **3a** in 50 mM HEPES buffer. Percentage area of starting material was determined after varying lengths of irradiation (302 nm) by LC-MS analysis. Data recorded in duplicate.*

Table S1. Photochemical properties of the photosensitisers used for screening.

Compound	Photosensitiser	E_T (kcal mol^{-1})	λ_{max} (nm)	τ (μs)
**PS1**	Ir(dF(CF$_3$)ppy)$_2$(dtbpy)	60.1[1]	380[2]	2.3[2]
**PS2**	Benzophenone	69.2[3]	335[4]	50[5]
**PS3**	Acetophenone	73.5[3]	335[3]	/
**PS4**	Xanthone	74.1[3]	610[3]	/
**PS5**	*N*-methylphthalimide	70.0[3]	560[3]	/
**PS6**	4,4'-Dimethoxybenzophenone	69.4[3]	545[3]	/
**PS7**	Thioxanthone	63.4[5]	625[3]	/

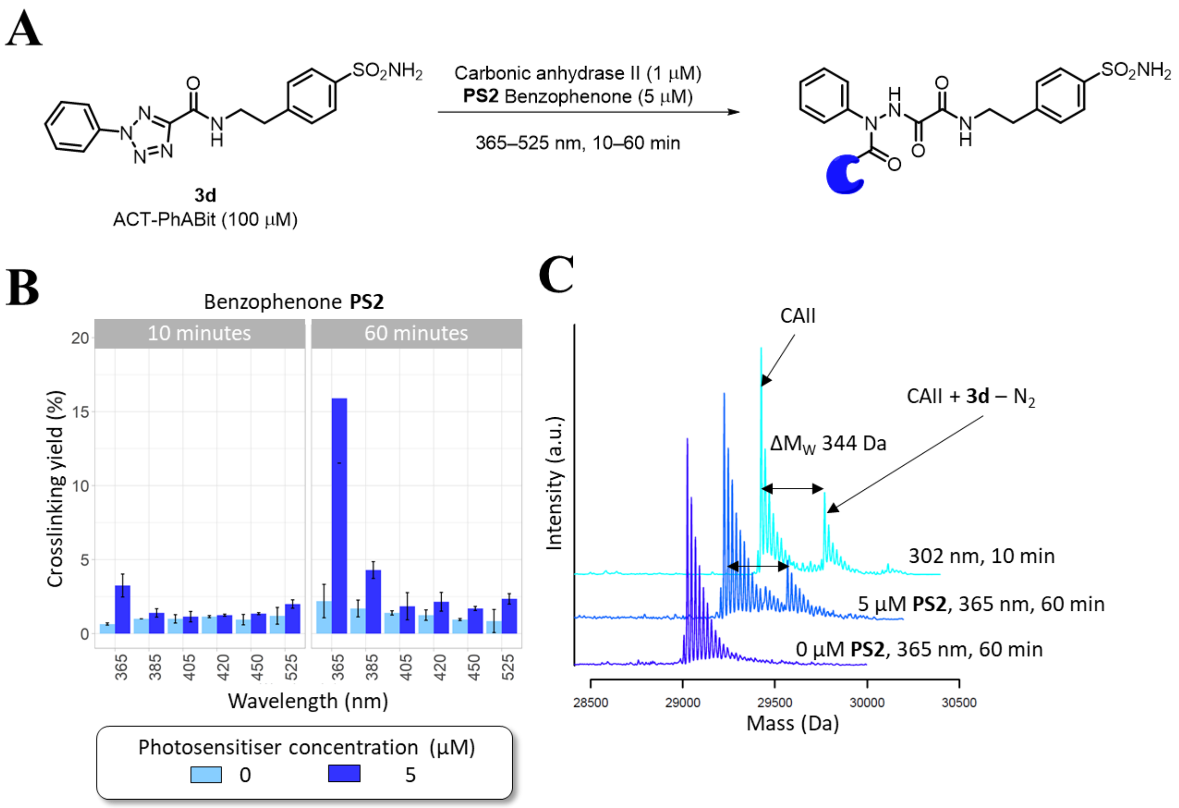

*Figure S8a) Photosensitiser screen. ACT-PhABit **3d** (100 µM) was irradiated (365–525 nm, 10–60 min) with carbonic anhydrase II (1 µM) in the presence of benzophenone (**PS2**, 5 µM) as a photosensitiser, in duplicate. b) Crosslinking yields at each wavelength screened. c) Mass spectra showing the benzophenone dependent crosslinking and comparison to direct activation with 302 nm light.*

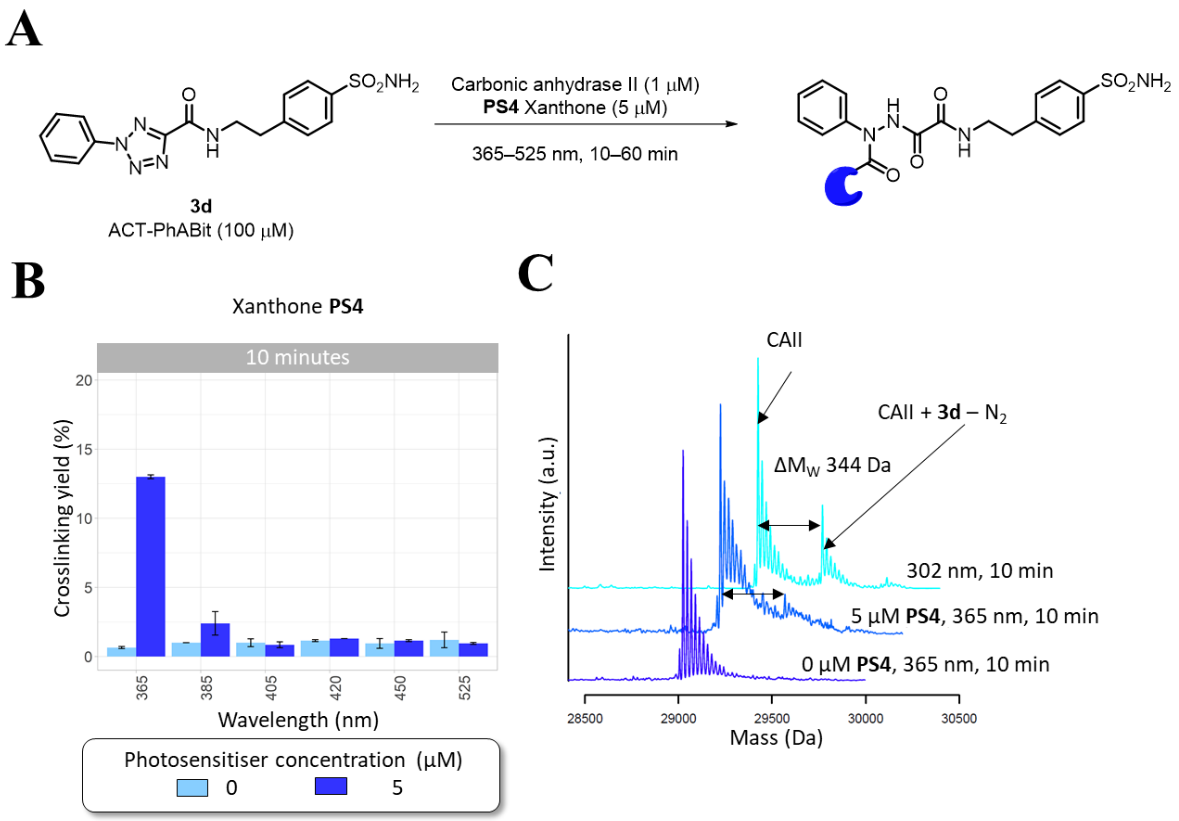

*Figure S9a) Photosensitiser screen. ACT-PhABit **3d** (100 µM) was irradiated (365–525 nm, 10–60 min) with carbonic anhydrase II (1 µM) in the presence of xanthone (**PS4**, 5 µM) as a photosensitiser, in duplicate. b) Crosslinking yields at each wavelength screened. c) Mass spectra showing the xanthone dependent crosslinking and comparison to direct activation with 302 nm light.*

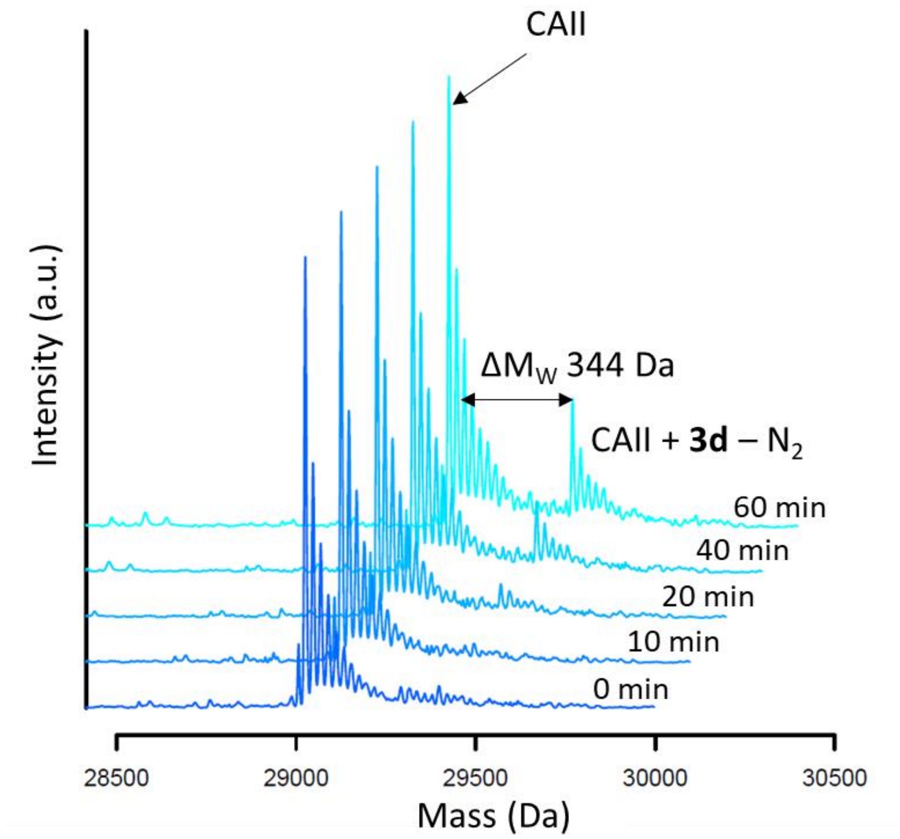

*Figure S10. Mass spectra overlay of ACT-PhABit **3d** (100 µM) irradiated (365 nm) with carbonic anhydrase II (1 µM) in the presence of benzophenone (**PS2**, 10 µM) for different lengths of time.*

2. General Methods

2.1 Solvents, reagents and consumables

Solvents were anhydrous and reagents purchased from commercial suppliers were used as received.

Protein stock solutions used:

- Carbonic anhydrase I from human erythrocytes (Sigma Aldrich, CAS: 9001-03-0, C4396-25MG, Lot: SLBT8230) supplied as a white powder (M_W 28781 Da)
- Carbonic anhydrase isozyme II from bovine erythrocytes (Sigma Aldrich, CAS: 9001-03-0, Lot: SLBV8282) supplied as a white powder (M_W 29024 Da)
- 6H-Tev-BRD4-BD1 (44–168) was produced as part of the GSK/GenScript collaboration and supplied as a solution (676 µM, M_W 15083 Da) in 10 mM HEPES, pH 7.5, with 100 mM NaCl
- KRAS4B-G12D-C118S (1–169) was produced as part of the GSK/GenScript collaboration and supplied as a solution (1550 µM, M_W 19344 Da) in 25 mM Tris, 100 mM NaCl, 5 mM $MgCl_2$, 1 mM TCEP, pH 8.0
- hBCL6-Flag-6H-Avi 3Cmut (5–129) was produced as part of the GSK/GenScript collaboration and supplied as a solution (352 µM, M_W 18357 Da) in 20 mM Tris, 250 mM NaCl, 5 mM DTT, 5% glycerol, pH 8.5
- Myoglobin from equine heart (Sigma Aldrich, CAS: 100684-32-02, Lot: SLBF8560V) supplied as a brown solid (M_W 16952 Da)

Buffer solutions used:

- Milli-Q phosphate-buffered saline (PBS) (pH 7.2)
- 50 mM HEPES (pH 7.4)

Plates used:

- Greiner 384 white low volume plates (#784075)
- Greiner 384 PP F-bottom plates (#781201)
- Labcyte ECHO Qualified 384LDV Plus (LPL-0200)

2.2 Irradiation

2.2.1 302 nm irradiation – general library screening

Irradiation was carried out using an Analytik Jena CL-1000 Ultraviolet Crosslinker (8 Watt, P/N 95-0230-02 with UV-B bulbs, P/N 34-0042-01) at 302 nm in Greiner 384 white low volume plates (#784075). Following irradiation plates were sealed with C.A.S plates seals (#12812447) prior to analysis.

2.2.2 Wavelength screening – photosensitiser studies

Irradiation was carried out using the Pacer Photochemistry LED Illuminator (POS0349-0100-AS-B) using the 24 LED array cassette (POS0358-0200-AS-A) (for full parts list see Table S2).[6] The array cassette featured 6 wavelengths (365, 385, 405, 420, 450, 525 nm), which allows irradiation in individual HPLC vials. Unless stated otherwise an LED current of 350 mA was used. The LEDs used in these arrays were manufactured by Ushio Opto Semiconductors Inc. They fall under the SMBB family, with an “02” lens, which focusses the output light within a narrow angle of 20°. The footprint of each LED is 5 mm × 5 mm.

Table S2. Pacer photochemical LED Illuminator part numbers

Pacer part number	Description
POS0349-0100-AS	Pacer Photochemistry LED Illuminator System. Includes driver panel, cables, TEC cooler, housing and vial holder
POS0358-0200-AS	24 LED array cassette, 6 wavelengths
POS0349-0110-AS	Control Panel
POS0349-0130-AS	Cooler Assembly
POS0349-0140-AS	Interconnecting Cable
POS0349-0150-AS	Cooler Power Cable
POS0349-0160-AS	Cooler Control Cable
POS0349-0810-AS	HPLC vial housing
POS0349-0400-AS	365 nm 48 LED Cassette
POS0349-0200-AS	385 nm 48 LED Cassette
POS0349-0500-AS	405 nm 48 LED Cassette
POS0349-0300-AS	420 nm 48 LED Cassette
POS0349-0600-AS	450 nm 48 LED Cassette
POS0349-0700-AS	525 nm 48 LED Cassette

2.3 Nuclear magnetic resonance (NMR) spectroscopy

Proton (^{1}H) and carbon (^{13}C) spectra were recorded in deuterated solvents at 30 °C (variable temperature (VT) spectra record high temperature spectra at 120 °C) using the standardised pulse methods on the Bruker AV-400/600 (^{1}H = 400/600 MHz, ^{13}C = 101/151 MHz, ^{19}F = 376 MHz) spectrometer. Chemical shifts are reported in parts per million (ppm) and referenced to tetramethylsilane (TMS) or the following solvent peaks: $CDCl_3$ (^{1}H = 7.27 ppm, ^{13}C = 77.0 ppm) or DMSO-d_6 (^{1}H = 2.50 ppm, ^{13}C = 39.5 ppm). Spectra were processed using ACD/Spectrus Processor (Version 2017.2). Peak assignments chosen based on chemical shifts, integrations and coupling constants, considering 2D analyses where necessary. Coupling constants are quoted to the nearest 0.1 Hz and multiplicities described as either singlet (s), doublet (d), triplet (t), quartet (q), quintet (quin), sextet (sxt), septet (sept), broad (br.) and multiplet (m).

2.4 Liquid chromatography-mass spectrometry (LC-MS) for small molecules

LC-MS was carried out on a Waters® Acquity UPLC instrument equipped with a CSH Acquity UPLC C18 column (internal diameter: 50 mm × 2.1 mm, packing diameter: 1.7 µm) at 40 °C with a 0.3 µL injection volume. The UV detection was a summed signal from wavelengths between 210 nm and 350 nm. Mass detection was performed with Alternate-scan Positive and Negative Electrospray on a Waters QDA instrument, with a scan range of 100–1000 Da and a scan frequency of 5 Hz.

Low pH: Sample was eluted using a gradient shown in Table S3 with a flow rate of 1.0 mL/min. Solvent A (0.1% *v/v* solution of formic acid in water) and solvent B (0.1% *v/v* solution of formic acid in acetonitrile).

Table S3. Low pH gradient for LC-MS analysis

**Time (min)**	**Flow Rate (mL/min)**	**Solvent A (%)**	**Solvent B (%)**
0.0	1	97	3
1.5	1	3	97
1.9	1	3	97
2.0	1	98	2

High pH: Sample was eluted using a gradient shown in Table S4 with a flow rate of 1.0 mL/min. Solvent A (10 mM ammonium bicarbonate in water adjusted to pH 10 with ammonia solution) and solvent B (acetonitrile).

Table S4. High pH gradient for LC-MS analysis

Time (min)	Flow Rate (mL/min)	Solvent A (%)	Solvent B (%)
0.0	1	100	0
0.05	1	100	0
1.5	1	3	97
1.9	1	3	97
2.00	1	100	0

2.5 In-plate LC-MS of ACT-PhABit library

For LC-MS of the ACT-PhABit library directly from the Greiner 384 PP F-bottom plate (#781201) the following protocol was used: LC-MS was carried out on a Waters® Acquity UPLC instrument equipped with a BEH Acquity UPLC C18 column (internal diameter: 50 mm × 2.1 mm, packing diameter: 1.7 µm) at 40 °C with a 0.2 µL injection volume. The UV detection was a summed signal from wavelengths between 210 nm and 350 nm. Mass detection was performed with Alternate-scan Positive and Negative Electrospray on a Waters SQD2 instrument, with a scan range of 100–1000 Da and a scan frequency of 5 Hz. A high pH (HpH) method was used and the sample was eluted using a gradient shown in Table S4 with a flow rate of 1.0 mL/min. Solvent A (10 mM ammonium bicarbonate in water adjusted to pH 10 with ammonia solution) and solvent B (acetonitrile).

2.6 Mass directed automated preparative HPLC (MDAP)

MDAP HPLC was carried out on a Waters® Xselect instrument equipped with a CSH C18 column (internal diameter: 150 mm × 30 mm, packing diameter: 5 µm) at ambient temperature with a 1 mL injection volume. The DAD detection was a summed signal from wavelengths between 210 nm and 350 nm. Mass detection was performed with Alternate-scan Positive and Negative Electrospray on a Waters QDA instrument, with a scan range of 100–1000 Da and a scan frequency of 4.3 Hz. Two procedures were used:

Low pH: C18 column (150 mm × 30 mm, 5 µm packing diameter, 40.0 mL/min flow rate) using a gradient elution at ambient temperature with the mobile phases of 0.1% *v/v* solution of formic acid in water (solvent A) and 0.1% *v/v* solution of formic acid in acetonitrile (solvent B).

High pH: C18 column (150 mm × 30 mm, 5 µm packing diameter, 40.0 mL/min flow rate) using a gradient elution at ambient temperature using mobile phases of water with 10 mM ammonium bicarbonate adjusted to pH 10 with ammonia solution (solvent A) and acetonitrile (solvent B).

The gradient of acetonitrile required to elute the product was determined by the LC-MS retention time. The methods were selected dependent on the retention time of desired material and are shown below (Table S5) and an exemplar gradient for Method B (Table S6).

Table S5. MDAP methods

Method	Flow rate (mL/min)	% Acetonitrile	LC-MS t_R (min)
A	40	0–30	0.40–0.65
B	40	15–55	0.65–0.90
C	40	30–85	0.90–1.16
D	40	50–99	1.16–1.40
E	40	80–99	1.40–2.00

Table S6. MDAP Method B solvent gradient

Time (min)	Flow rate (mL/min)	Solvent A (%)	Solvent B (%)
0.0	40	85	15
3.0	40	85	15
12.0	40	45	55
12.5	40	45	55
13.0	40	1	99
17.0	40	1	99

2.7 Infrared Spectroscopy

IR spectra were recorded using a PerkinElmer® Spectrum Two FT-IR machine. Absorption maxima (ν_{max}) are reported in wavenumbers (cm^{-1}) for peaks outside of the fingerprint region.

2.8 Melting Point

Melting points were recorded on a BUCHI Melting Point M-565 apparatus.

2.9 High-resolution mass spectrometry (HRMS)

HRMS were recorded on a Waters® Acquity UPLC instrument equipped with a BEH (HpH method)/CSH (low pH method) C18 column (internal diameter: 100 mm × 2.1 mm, packing diameter: 1.7 µm) at 50 °C with a 0.2 µL injection volume. The UV detection was a summed signal from wavelengths between

210 nm and 500 nm. Mass detection was performed with Alternate-scan Positive and Negative Electrospray on a Waters XEVO G2-XS QToF instrument, with a scan range of 100–1200 Da. Two procedures were used:

Low pH: Gradient elution using mobile phases of water with 0.1% *v/v* solution of formic acid (solvent A) and 0.1% *v/v* solution of formic acid in acetonitrile (solvent B) (Table S7).

High pH: Gradient elution using mobile phases of water with 10 mM ammonium bicarbonate adjusted to pH 10 with ammonia solution (solvent A) and acetonitrile (solvent B) (Table S8).

Table S7. HRMS low pH method solvent gradient

Time (min)	Flow rate (mL/min)	Solvent A (%)	Solvent B (%)
0.0	0.8	95	5
8.5	0.8	7	93
9.0	0.8	7	93
9.5	0.8	95	5
10.0	0.8	95	5

Table S8. HRMS HpH method solvent gradient

Time (min)	Flow rate (mL/min)	Solvent A (%)	Solvent B (%)
0.0	0.8	99	1
0.5	0.8	99	1
17.0	0.8	10	90
18.5	0.8	10	90
19.0	0.8	99	1
20.0	0.8	99	1

2.10 TLC and Flash column chromatography

TLC was carried out using polyester-backed precoated silica plates (0.2 mm particle size). Spots were visualised under UV light of λ_{max} = 254 nm or 365 nm or by heating with potassium permanganate stain.

Column chromatography was carried out using the Teledyne ISCO Combi*Flash*® R*f+* apparatus with Redi*Sep*® silica cartridges. Compounds were visualised by UV (λ_{max} = 254 and 280 nm).

2.11 Intact-protein LC-MS

Intact-protein masses were recorded by LC-MS using an Agilent G6230B time-of-flight (ToF) Accurate Mass Series mass spectrometer, interfaced with an Agilent 1290 infinity II series column oven (G7116B) and an Agilent 1290 infinity II series liquid chromatography high speed binary pump (G7120A). The protein sample was injected using an Agilent 1290 infinity II series multisampler with dual needles (Model No. G7167B) with a 10 µL injection volume and maintained at a temperature of 4 °C. Chromatography was carried out on an Agilent Bio-HPLC PLRP-S (1000 Å, 5 µm × 50 mm × 1.0 mm, PL1312-1502) reverse phase HPLC column at 70 °C. The sample was eluted at 0.5 mL/min using a gradient system from Solvent A (water, 0.2% (*v/v*) formic acid) to Solvent B (acetonitrile, 0.2% (*v/v*) formic acid) according to the conditions described in Table S9. The eluent was injected directly into an Agilent ToF mass spectrometer (Model No. G6230B) using a dual AJS ESI source and scanning between 600–3200 Da with a scan rate of 1.20 s in positive mode. The following MS parameters were used: capillary voltage limit – 4000 V; desolvation temperature – 350 °C; drying gas flow – 10 L/min.

Table S9. Intact-protein LC-MS solvent gradient

Time (min)	Flow rate (mL/min)	Solvent A (%)	Solvent B (%)
0.6	0.5	80	20
0.61	0.5	50	50
1.0	0.5	0	100
1.2	0.5	0	100
1.21	0.5	80	20

Data acquisition was carried out in 2 GHz Extended Dynamic range mode. Spectra were processed using Mass Hunter Qualitative Analysis™ B06.00 (Agilent) software with the Maximum Entropy method employed. The total ion chromatograms (TIC) were extracted (region containing protein) and the summed scans were deconvoluted (using a maximum entropy algorithm) over a mass to charge ratio (*m/z*) range dependent on the protein (Table S10).

Table S10. Deconvolution conditions for target proteins examined in this work

**Protein**	***m/z* range**	**Expected mass range (Da)**
Carbonic Anhydrase I	800–2000	27000–32000
Carbonic Anhydrase II	800–2000	27000–31000
BRD4-BD1	800–2000	13000–17000
$KRAS^{G12D}$	800–2000	17000–21000
BCL6	800–2000	15000–20000
Myoglobin	800–2000	15000–18000

The deconvoluted spectra were exported as csv files and analysed using R Studio software (Version 3.6.3) to generate pdf files of the spectra. The median of the protein only controls were subtracted from the sample spectra to remove baseline signal. The peak height for unmodified protein and labelled protein were recorded and used to calculate percentage photocrosslinking using the equation:

$$\% \, crosslinking = \frac{height \; of \; modification \; peak}{height \; of \; modification \; peak + height \; of \; parent \; peak} \times 100$$

2.12 Centrifuge

Plates were centrifuged using a Sorvall Legend RT (401198833) model at 1000 rpm for 1 minute, prior to sampling.

2.13 Protein MOE calculations

X-ray structures for the six proteins screened were imported into MOE (Version 2019.0101). The associated PDB codes are shown in Table S11. The structures were prepared using the 'QuickPrep' tool and ligands were removed, before the protein properties were calculated with the following parameters: pH 7.4, 300 K, 0.1 M salt concentration. This enabled the determination of solvent exposed surface area of aspartate and glutamate residues.

Table S11. Protein data bank (PDB) codes for the proteins that were screened against the ACT-PhABit library

Protein	PDB
Carbonic Anhydrase I	1AZM
Carbonic Anhydrase II	1V9E
BRD4-BD1	3UVW
KRAS^{G12D}	5US4
BCL6	1R29
Myoglobin	1WLA

3. Library synthesis

3.1 Amine selection

Amines were selected from our previously reported 1073-membered HTC-PhABit library.[7] All amines were alkylamines and contained only one amine group. The structures had been tagged based on 6 aliphatic amine types, differentiating based on primary/secondary, hindered/non-hindered (hindered: ≥2 substituents α to nitrogen) and cyclic/non-cyclic. Based on our previous findings, primary unhindered, primary hindered and secondary cyclic unhindered amines achieved the highest conversions in the HTC reactions and so only amines with these annotations were chosen, the rest were filtered out.[7] A small number (<5) of positive controls were also included which belonged to the one other categories of amines (*e.g.* secondary cyclic hindered, **3e**). This left a selection of 546 amines which were available from GSK compound stores.

From our previous report[7] these selected amines also fulfilled the following criteria:

- Unstable compounds and compounds with other liabilities had been removed using proprietary GSK filters
- $150<M_W<250$
- cLogP<9 (BioByte)[8]
- p*K*a<7 (ChemAxon)[9]

The selected amines (10 mM, 20 µL) were ordered from GSK's solution stores in Greiner 384 PP F-bottom plates (#781201). Following small molecule LC-MS directly from the plates to assess the amine's initial purity the HTC reactions were undertaken (**SI section 2.5**).

3.2 ACT-PhABit synthesis

Two Greiner PP 384-well plates were charged with 546 amines (10 mM, 16.5 µL). LC-MS analysis of the amine starting materials was conducted prior to reaction (**SI section 2.5**). A stock solution of OSu ester **1** and NEM was prepared in DMSO such that the respective concentrations were 44 and 120 mM. To the plated amines, the stock solution (4.1 µL) was added, meaning the final concentration of the ACT-PhABits were 8 mM in 20.6 µL. The plates were sealed, centrifuged (1 min, 1000 rpm) and left standing at room temperature for 24 h. After the reaction the plates were subjected to small molecule LC-MS analysis to identify and quantify product formation (**SI section 2.5**).

To quench the reaction mixture, a 5.2 µL aliquot of the reaction mixture was removed and added to new Labcyte 384LDV plates (LPL-0200). A hydroxylamine solution (50% wt in H_2O, 30.3 M) was diluted 1 in 10 to afford a 3.03 M hydroxylamine solution which was dispensed (5 µL) across an empty Labcyte 384LDV plate (LPL-0200). Using a Labcyte ECHO© 555 Liquid Handler, 30 nL of the hydroxylamine solution was added to each reaction mixture well such that its final concentration was 17.6 mM. The plates were sealed and left to stand at room temperature for 1 h. The plates were stored in a –20 °C freezer.

4. Screening the ACT-PhABit library

4.1 ACT-PhABit plate preparation

Using a Labcyte ECHO© 555 Liquid Handler 190 nL of the ACT-PhABits (8 mM) and 110 nL of DMSO were transferred to new Greiner 384 white low volume plates (#784075). Such that the ACT-PhABit concentration was 5 mM in 300 nL prior to protein addition.

4.2 Protein addition and irradiation protocol

Protein samples were prepared for screening according to the following protocols:

- For solid proteins (carbonic anhydrase I (CAI), carbonic anhydrase II (CAII) and myoglobin), the solid was weighed out (typically ~1–1.5 mg) and dissolved in the appropriate volume of buffer (PBS or HEPES) such that its concentration was 100 µM. This stock solution was then further diluted in either PBS/HEPES to afford a 1 µM protein solution.
- For dissolved protein samples (BRD4-BD1, $KRAS^{G12D}$, BCL6), the stock solution was diluted in the appropriate volume of buffer (PBS or HEPES) such that the concentration was 1 µM.

To the compound containing (300 nL, 5 mM) Greiner 384 white low volume plates (#784075) a 15 µL aliquot of protein stock solution (1 µM) was added to the ACT-PhABit containing wells at 4 °C, meaning the final DMSO composition was 2.0% (*v/v*) and the final concentration of ACT-PhABit was 100 µM. To the control and blank wells respectively, protein stock solution (15 µL, 1 µM) and MQ water (15 µL) were added. The plate was equilibrated at 4 °C for 15 minutes before irradiation with UV light (302 nm, **SI section 2.2.1**) for 10 minutes. The plates were centrifuged (1 min, 1000 rpm). The individual wells were analysed by LC-MS-ToF mass spectrometry (**SI section 2.11**).

4.3 Screening the 546-membered ACT-PhABit library

The library of 546 ACT-PhABits were plated according to **SI section 4.1** and a 1 µM stock solution of each protein stock solution (PBS/HEPES, Table S12) was prepared and added across the plates (**SI section 4.2**). The plate was then subjected to UV irradiation (**SI section 2.2.1**). Protein concentration: 1 µM, ACT-PhABit concentration: 100 µM, DMSO content 2.0% (*v/v*). The individual wells of the 2 × 384-well plates were analysed by LC-MS-ToF mass spectrometry (**SI section 2.11**). Hits for each protein were classed as those ACT-PhABits displaying a baseline subtracted crosslinking yield >mean + 2SD %. The 6 proteins were screened either in PBS of HEPES buffer according to Table S12 below:

Table S12. Buffer used for single shot screen of ACT-PhABit versus each protein

**Protein**	**Buffer**
Carbonic Anhydrase I	PBS/HEPES
Carbonic Anhydrase II	HEPES
BRD4-BD1	HEPES
$KRAS^{G12D}$	PBS/HEPES
BCL6	HEPES
Myoglobin	HEPES

5. Follow-up studies: photolysis, competition and LC-MS/MS

5.1 ACT-photolysis studies

5.1.1 ACT photolysis product identification

A 10 mM solution of ACT-PhABit **3d** (1.0 mg, 2.69 µmol) in DMSO was prepared. The solution was diluted (2 µL) in 50 mM HEPES (200 µL) such that the final concentration **3d** was 100 µM (DMSO 1.0% *v/v*). The resultant solution was irradiated (302 nm, **SI section 2.2.1**) for 10 min and sampled by LC-MS (**SI section 2.4**). Products were identified from the corresponding masses of detected product LC-MS peaks. No material was isolated.

5.1.2 Rate of photolysis of ACT-PhABit **3a** by ^{1}H NMR

A solution of ACT-PhABit **3a** (1.1 mg, 2.71 µmol) was dissolved in 7:3 DMSO-*d*6:D_2O (1 mL). To the solution was added internal standard, dimethylsulfone (2.7 mg, 0.03 mmol). The resultant solution was irradiated (302 nm, **SI section 2.2.1**) for 10 min in a 384-well plate taking time points at 0, 0.5, 1, 2.5, 5, 10 min for ^{1}H NMR analysis. The molar ratio of the starting material **3a** relative to the internal standard was determined to elucidate the photolysis rate. The experiment was repeated (n = 2) and the mean rate of photolysis was plotted. No material was isolated.

5.1.3 Rate of photolysis of ACT-PhABit **3a** by LC-MS

A solution of ACT-PhABit **3a** (0.8 mg, 1.97 µmol) was dissolved in DMSO (99 µL) to afford a 20 mM solution. The solution was further diluted (2 µL) in 50 mM HEPES (198 µL) to afford a 200 µM solution, 1.0% DMSO (*v/v*). The resultant solution was irradiated (302, **SI section 2.2.1**) for 10 min, in duplicate, taking time points at 0, 0.5, 1, 2.5, 5, 10 min for LC-MS analysis (**SI section 2.4**). The mean LC-MS % area for the starting material (**3a**) and was recorded was plotted. No material was isolated.

5.2 KRAS^{G12D} competition studies with BI-2852

ACT-PhABit **4a** was dissolved in DMSO at a final concentration of 10 mM. The 10 mM ACT-PhABit stock solution was added (5 µL) into a Labcyte ECHO Qualified 384LDV Plus plate (LPL-0200). Using a Labcyte ECHO© 555 Liquid Handler the ACT-PhABit was diluted with DMSO (in triplicate) (75 nL + 75 nL) such

that its concentration was 5 mM (150 nL) in a Greiner 384 white low volume plate. A 1 µM $KRAS^{G12D}$ stock was prepared in HEPES buffer (**SI section 4.2**). The protein solution was split into 2 × 2 mL portions and to one was added competitor (BI-2852, 5 mM, 2 µL)[10] such that its final concentration was 5 µM, while to the second was added DMSO (2 µL). To the compound in the Greiner 384 white low volume plate was added either a 15 µL aliquot of protein stock solution (1 µM) + competitor (5 µM) or protein stock solution (1 µM) + DMSO at 4 °C, meaning the final DMSO composition was 1.1 % (*v/v*) and the final concentration of the ACT-PhABit was 50 µM. To the control and blank wells respectively, protein stock solution (15 µL, 1 µM) and MQ water (15 µL) were added. The plate was equilibrated at 4 °C for 15 minutes before irradiation with UV light (302 nm, **SI section 2.2.1**) for 10 minutes. The plate was centrifuged (1 min, 1000 rpm). The individual wells were analysed by LC-MS-ToF mass spectrometry (**SI section 2.11**).

5.3 Site of crosslinking LC-MS/MS studies

Solutions of selected ACT-PhABit hits (**4a**, **5a**, 100 µM) in HEPES were prepared with either $KRAS^{G12D}$ or BCL6 (3 µM) according to **SI section 4**. The solutions were incubated and irradiated according to **SI section 4**. Un-irradiated control samples were also prepared in the same way for each ACT-PhABit.

Irradiated and non-irradiated samples for each compound were processed for LC-MS/MS analysis. Samples (1 µg) were separated by 4–12% bis-tris sodium dodecyl sulfate-polyacrylamide gel electrophoresis (SDS-PAGE) using 2-(*N*-morpholino)ethanesulfonic acid (MES) running buffer (ThermoFisher) to remove the excess of unbound compound alongside a SeeBlue protein ladder. Gel was stained with colloidal Comassie InstantBlue (Expedeon) and bands corresponding to $KRAS^{G12D}$ or BCL6 were excised and transferred to LoBind microtubes (Eppendorf). Gel pieces were destained by addition of 400 µL of ammonium bicarbonate pH 8.0/acetonitrile (1:1 *v:v*, Sigma Aldrich) and agitated for 30 min at room temperature. The destain solution was discarded and the samples were reduced by addition of 100 µL of 10 mM TCEP (in 100 mM ammonium bicarbonate pH 8.0, Thermo Fisher) for 30 min at 65 °C. The reducing solution was discarded and samples were alkylated by addition of 100 µL of 10 mM iodoacetamide (in 100 mM ammonium bicarbonate pH 8.0, Sigma Aldrich) for 30 min at room temperature in the dark. Samples were washed sequentially with 400 µL of 25 mM ammonium bicarbonate pH 8.0, 400 µL of 25 mM ammonium bicarbonate pH 8.0/acetonitrile (1:1, *v:v*) and 400 µL of acetonitrile with brief agitation between each wash step. Samples were digested overnight at 37 °C with trypsin/LysC (Promega) at a 1:5 enzyme to sample ratio in 40 µL of 25 mM ammonium bicarbonate pH 8.0. The resulting supernatant was transferred to new LoBind microtubes and peptides were extracted by washing the gel pieces with 50 µL of acetonitrile and brief vortexing. This was repeated twice and pooled with the supernatant. The extracted peptide solutions were snap frozen

on dry ice before being vacuum dried (Eppendorf Speedvac). Dried peptides were resuspended in 0.1% formic acid, 0.05% trifluoroacetic acid, agitated briefly before centrifugation (4000 × g, 5 min) and transferred to autosampler vials (Waters).

Digested samples were injected on an Easy-nLC 1000 UHPLC system (Thermo Scientific). The nanoLC was interfaced to a Q-Exactive Hybrid Quadrupole-Orbitrap Mass Spectrometer (Thermo Scientific). Tryptic peptides were loaded on a 2 cm × 75 µm Acclaim PepMap 100 C18 trapping column (Thermo Scientific) and separated on a 25 cm × 75 µm, 2 µm particles, PepMap C18 EasySpray analytical column (Thermo Scientific) using a 50 min gradient of 2–38% acetonitrile, 0.1% formic acid and a flow rate of 300 nL/min. LC-MS/MS based peptide sequencing was first performed by data dependent analysis (DDA) as follows: full MS 400–1600 *m/z* at 70,000 resolution, MS AGC target 1e6, MS Maximum IT 200 ms, followed by MS/MS top 10 HCD fragmentation, with stepped normalised collision energy (CE) 23, 27 and 30 V, isolation window 1.5 *m/z*, fixed first mass 145 *m/z*, 17,500 resolution, MS/MS AGC target 5e4 and MS/MS Maximum IT 200 ms.

When necessary to assign the exact site of modification within a modified peptide, samples were also analysed using Parallel Reaction Monitoring (PRM) methods. Full MS settings were as above, PRM parameters were resolution 17,500, AGC target 2e5, maximum IT 200 ms, isolation window 1.2 *m/z*, fixed first mass 145 *m/z*. An inclusion list was generated for each experimental method targeting the peptide of interest. For $KRAS^{G12D}$ + compound **4a** the following *m/z* values were used: peptide 151–162 2+ ion (*m/z* 692.3488, CE 30 and 32 V) and 3+ ion (*m/z* 461.9015, CE 27 and 30 V) and peptide 151–162 modified by compound **4a** 2+ ion (*m/z* 870.9037 CE 30 and 32 V) and 3+ ion (*m/z* 580.9387, CE 27 and 30 V). For BCL6 + compound **5a**: peptide 98–121 3+ ion (*m/z* 940.0824, CE 30 or 32 V) and 4+ ion (*m/z* 705.3136, CE 27 or 30 V) and peptide 98–121 modified by compound **5a** 4+ ion (*m/z* 798.8506, CE 27 or 30 V).

Uninterpreted tandem MS spectra were searched for peptide matches against the $KRAS^{G12D}$ and BCL6 sequences using the Mascot software (Version 2.6.0) (Matrix Science) with a 5 ppm mass tolerance for peptide precursors and 20 mDa mass tolerance for fragment ions and trypsin/P as the cleavage enzyme with up to 2 missed cleavages allowed. Oxidation on methionine, carbamidomethylation on cysteine and ACT-PhABit – N_2 modification on aspartate and glutamate were allowed as variable modifications. Samples were also searched with ACT-PhABit – N_2 modification with no amino acid preference to confirm glutamate and aspartate preference (data not shown). PRM MS/MS spectra were manually validated and annotated.

6. ACT photosensitiser studies

6.1 Photosensitiser screen

Separate stock solutions of the 7 × photosensitisers (**PS1**–**7**, Sigma Aldrich, Table S1) in DMSO (5 mM) and ACT-PhABit **3d** in DMSO (10 mM) were prepared. A 100 µM solution of CAII in PBS was prepared according to **SI section 4.2**, which was further diluted (10 µL) in 1 mL PBS to afford a 1 µM CAII solution, this was repeated 9 times (7 × **PS**, 1 × –**PS** control, 1 × protein only control). *N.B.* PBS used due to fault with MS caused HEPES adducts which confounded analysis. To 7 of the 1 µM CAII solutions was added each of the photosensitisers (1 µL) such that the final concentration of each photosensitiser was 5 µM. To the 8th CAII solution was added 1 µL DMSO to act as a negative control (–**PS**). Finally, to each of the 8 CAII solutions was added 10 µL of the ACT-PhABit **3d** stock (10 mM) such that the final concentration of ACT-PhABit **3d** was 100 µM (1.1% DMSO content (*v/v*)). To the 9th CAII solution was added DMSO (11 µL) to act as the protein only control (1.1% DMSO content (*v/v*)).

For the irradiation, a 50 µL portion of each solution of CAII (1 µM) + ACT-PhABit **3d** (100 µM) + **PS1**–**7** (5 µM) was added to LC-MS vials (in duplicate) which were and left to equilibrate at 4 °C for 15 minutes before irradiation with light (365, 385, 405, 420, 450, 525 nm, 350 mA). The Pacer photochemical LED Illuminator 24 LED (6 wavelength array) was used as the light source (**SI section 2.2.2**). At 10 and 60 minute time-points a 15 µL sample was taken from each vial and entered into a Greiner white LDV 384-well plate (#784075). To the control and blank wells respectively, protein stock solution (15 µL, 1 µM, 1.1% DMSO *v/v*) and MQ water (15 µL) were added. The plate was centrifuged (1 min, 1000 rpm). The individual wells were analysed by LC-MS-ToF mass spectrometry (**SI section 2.11**).

The crosslinking versus wavelength ±photosensitiser at 10 and 60 minutes was plotted using R Studio software (Version 3.6.3, ggplot), each data point was recorded in duplicate and the mean and SD were calculated and plotted.

6.2 Benzophenone photosensitiser follow-up timecourse

Stock solutions of benzophenone (**PS2**) in DMSO (10 & 5 mM) and ACT-PhABit **3d** in DMSO (10 mM) were prepared. Separate solutions of CAII (1 µM) + ACT-PhABit **3d** (100 µM) + benzophenone (10, 5, 0 µM) were prepared in PBS according to **SI section 6.1** (1.1% DMSO content (*v/v*)), along with protein-only controls.

For the irradiation step, a 200 µL portion of each solution of CAII (1 µM) + ACT-PhABit **3d** (100 µM) + benzophenone (10, 5, 0 µM) was added to LC-MS vials (in duplicate) which were and left to equilibrate at 4 °C for 15 minutes before irradiation with light (365 nm, 350 mA). The Pacer photochemical LED illuminator 24 LED (6 wavelength array) was used as the light source (**SI section 2.2.2**). At 0, 10, 20, 40 and 60 minute time points a 15 µL sample was taken from each vial and entered into a Greiner white LDV 384-well plate (#784075). For comparative purposes ACT-PhABit **3d** (100 µM) was also irradiated with CAII (1 µM) at 302 nm (10 min, **SI section 2.2.1**) (1.1% DMSO content (*v/v*)) in PBS (15 µL), in duplicate, and entered into the plate. To the control and blank wells respectively, protein stock solution (15 µL, 1 µM, 1.1% DMSO *v/v*) and MQ water (15 µL) were added. The plate was centrifuged (1 min, 1000 rpm). The plate was centrifuged (1 min, 1000 rpm). The individual wells were analysed by LC-MS-ToF mass spectrometry (**SI section 2.11**). The crosslinking versus time at each photosensitiser concentration was plotted on GraphPad Prism 5.0.4, each data point was recorded in duplicate and the mean and SD were calculated and plotted.

6.3 Protein intensity studies

To compare the effect of ±irradiation on protein, CAII (1 µM) was irradiated in PBS (1.1% DMSO *v/v*) under the following conditions:

- 302 nm, 10 mins
- 365 nm, 60 mins, benzophenone (10 µM)
- No irradiation

Protein signal intensity was recorded by the MS and processed using R Studio software (Version 3.6.3) before plotting in excel.

7. Compounds

7.1 Synthesis of ACT-OSu ester **1**

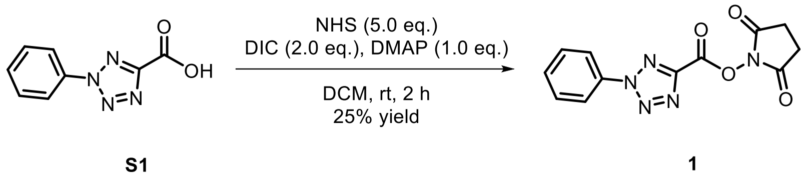

Scheme S1

2,5-dioxopyrrolidin-1-yl 2-phenyl-2*H*-tetrazole-5-carboxylate (1)

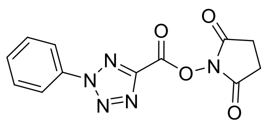

A solution of 2-phenyl-2*H*-tetrazole-5-carboxylic acid (**S1**) (100 mg, 0.53 mmol), 1-hydroxypyrrolidine-2,5-dione (NHS) (303 mg, 2.63 mmol), *N,N'*-Diisopropylcarbodiimide (DIC) (0.16 mL, 1.05 mmol) and *N*,*N*-dimethylpyridin-4-amine (64 mg, 0.53 mmol) in CH_2Cl_2 (10 mL) was stirred at rt for 2 h. The reaction mixture was filtered over cotton wool. The resultant solution was concentrated in vacuo to afford a yellow solid. The solid was suspended in CH_2Cl_2 before filtering and loading onto a silica column. The mixture was purified by flash column chromatography (silica, 0–100% EtOAc in cyclohexane). The relevant fractions were concentrated in vacuo to afford an off-white solid. The solid was dried under a stream of nitrogen at rt for 1 h. The product was further dried in a vacuum oven, heated at 40 °C, for 16 h to afford 2,5-dioxopyrrolidin-1-yl 2-phenyl-2*H*-tetrazole-5-carboxylate (**1**) (38 mg, 0.13 mmol, 25 % yield) as an off-white solid. Infrared spectrum: ν_{max} (solid): 2967, 1820, 1731, 1209, 1108, 993, 766, 669, 647 cm^{-1}. ^{1}H NMR (400 MHz, $CDCl_3$) δ = 8.19–8.25 (m, 2H), 7.56–7.66 (m, 3H), 2.96 (s, 4H) ppm. ^{13}C NMR (151 MHz, $CDCl_3$) δ = 168.1, 154.0, 153.5, 136.2, 131.2, 130.0, 120.5, 25.7 ppm.

7.2 General procedure for the re-synthesis of ACT-PhABit hits

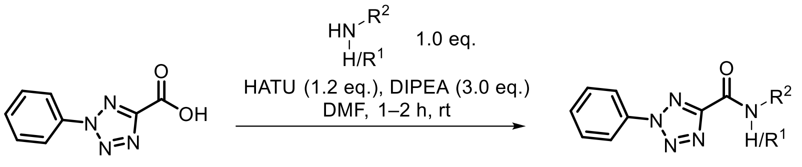

S1
1.0 eq.

3a, **3d**, **5a**

Scheme S2

A solution of compound **S1** (1.0 eq.), HATU (1.2 eq.) and DIPEA (3.0 eq.) in DMF (1 mL) was stirred at rt for 0.25 h. At which point an amine (1.0 eq., 0.11–0.16 mmol) was added and the resultant solution was stirred at rt for a further (1–2 h). The reaction mixture was purified by MDAP (HpH_Method_B). The relevant fraction(s) were combined and concentrated in vacuo. The products were dried in a vacuum oven, heated at 40 °C, for 6 h to afford the desired products.

7.3 ACT-PhABit hits

***N*-(2-chloro-6-methyl-4-sulfamoylbenzyl)-2-phenyl-2*H*-tetrazole-5-carboxamide (3a)**

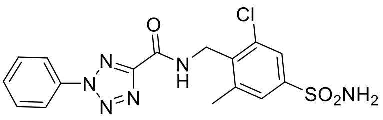

Following the general procedure outlined in **SI section 7.2** afforded *N*-(2-chloro-6-methyl-4-sulfamoylbenzyl)-2-phenyl-2*H*-tetrazole-5-carboxamide (**3a**) (15 mg, 0.04 mmol, 32%) as a white solid. Infrared spectrum: ν_{max} (solid): 3353, 1685, 1547, 1340, 1180, 1148, 763, 622 cm^{-1}. ^{1}H NMR (400 MHz, DMSO-d_6) δ = 9.43 (br t, *J* = 5.5 Hz, 1H), 8.09–8.14 (m, 2H), 7.60–7.74 (m, 5H), 7.45 (br s, 2H), 4.72 (br d, *J* = 5.5 Hz, 2H), 2.53 (s, 3H) ppm. ^{13}C NMR (151 MHz, DMSO-d_6) δ = 159.7, 156.2, 144.2, 141.4, 136.9, 135.9, 134.9, 130.7, 130.2, 125.8, 123.9, 120.3, 38.2, 20.0 ppm. LC-MS (CSH~2min_HpH) t_r = 0.96 min, $[M–H]^-$ = 405.13, (96% purity). HRMS: ($C_{16}H_{15}N_6O_3S$) $[M+H]^+$ requires 407.0615, found $[M+H]^+$ 407.0690.

2-phenyl-*N*-(4-sulfamoylphenethyl)-2*H*-tetrazole-5-carboxamide (3d)

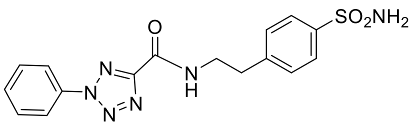

Following the general procedure outlined in **SI section 7.2** afforded 2-phenyl-*N*-(4-sulfamoylphenethyl)-2*H*-tetrazole-5-carboxamide (**3d**) (13 mg, 0.04 mmol, 22%) as a white solid. Infrared spectrum: ν_{max} (solid): 3325, 1675, 1543, 1305, 1156, 761, 683, 598 cm^{-1}. ^{1}H NMR (400 MHz, DMSO-d_6) δ = 9.25 (br t, *J* = 6.6 Hz, 1H), 8.10–8.15 (m, 2H), 7.74–7.78 (m, 2H), 7.62–7.73 (m, 3H), 7.43–7.48 (m, 2H), 7.27 (s, 2H), 3.61 (dt, *J* = 6.6, 6.6 Hz, 2H), 2.99 (t, *J* = 6.6 Hz, 2H) ppm. ^{13}C NMR (151 MHz, DMSO-d_6) δ = 159.9, 156.1, 143.3, 142.1, 136.0, 130.7, 130.2, 129.1, 125.7, 120.3, 40.1, 34.4 ppm. LC-MS (CSH~2min_HpH) t_r = 0.86 min, $[M–H]^-$ = 371.18, (100% purity). HRMS: ($C_{16}H_{16}N_6O_3S$) $[M+H^+]$ requires 373.1005, found $[M+H^+]$ 373.1079

2-(1-(2-phenyl-2*H*-tetrazole-5-carbonyl)pyrrolidin-3-yl)-1*H*-benzo[d]imidazole-7-carboxamide (5a)

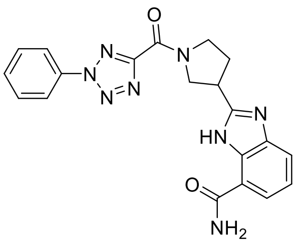

Following the general procedure outlined in **SI section 7.2** afforded 2-(1-(2-phenyl-2*H*-tetrazole-5-carbonyl)pyrrolidin-3-yl)-1*H*-benzo[d]imidazole-7-carboxamide (**5a**) (11 mg, 0.03 mmol, 17%) as a pale yellow solid. Infrared spectrum: ν_{max} (solid): 2973, 1644, 1606, 1491, 1414, 1054, 1033, 1007, 754 cm^{-1}. ^{1}H NMR (400 MHz, DMSO-d_6, 120 °C) δ = 12.42 (br s, 2H) 8.07–8.17 (m, 2H) 7.82 (br d, J = 8.07 Hz, 1H) 7.59–7.76 (m, 4H) 7.26 (t, J = 8.07 Hz, 1H) 4.08–4.46 (m, 3H) 3.75–4.00 (m, 2H) 2.38–2.49 (m, 2H) ppm. ^{13}C NMR (151 MHz, DMSO-d_6, rotamers observed, not all signals found) δ = 160.2, 160.1, 155.8, 155.8, 136.0, 130.7, 130.2, 120.4, 47.9, 46.3, 31.2, 29.1 ppm. LC-MS (CSH~2min_HpH) t_r = 0.84 min, $[M+H]^+$ = 402.97, (100% purity). HRMS: ($C_{20}H_{18}N_8O_2$) $[M+H]^+$ requires 403.1553, found $[M+H]^+$ 403.1626.

7.4 Synthesis of KRAS^{G12D} hit **4a**

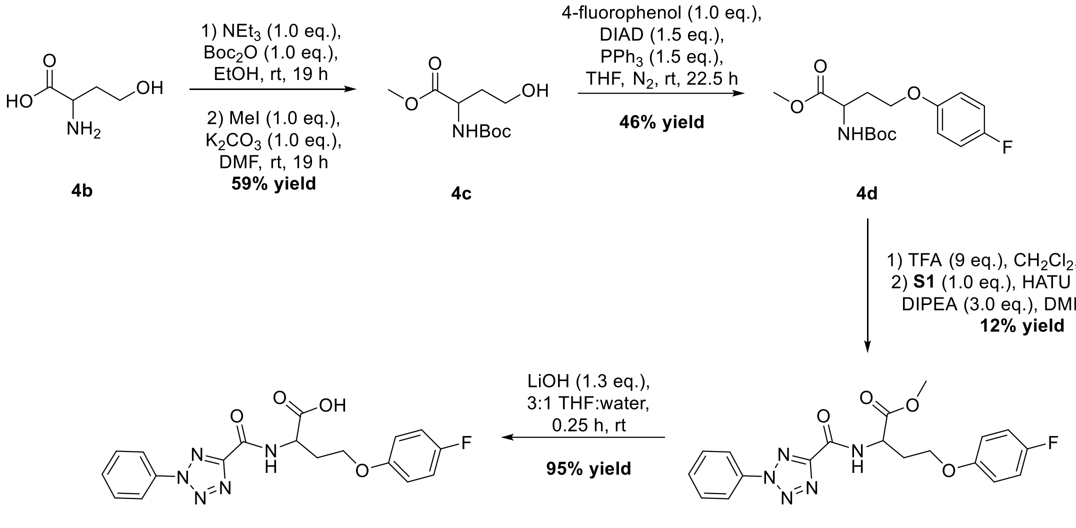

Scheme S3

methyl (tert-butoxycarbonyl)homoserinate (4c)

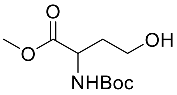

A solution of D/L-homoserine (**4b**) (500 mg, 4.20 mmol), triethylamine (0.59 mL, 4.20 mmol) and Boc_2O (0.98 mL, 4.20 mmol) in EtOH (6 mL) was stirred at rt for 19 h. The solution was concentrated in vacuo to afford a white liquid. The liquid was stirred in DMF (5 mL) and K_2CO_3 (580 mg, 4.20 mmol) and iodomethane (0.26 mL, 4.20 mmol) were added. The resultant solution was stirred at rt for 19 h. The reaction mixture was quenched with sat. aq. NH_4Cl (20 mL) and extracted with CH_2Cl_2 (3 × 40 mL). The combined organic layers were dried (hydrophobic frit) before concentration in vacuo to afford a colourless liquid. The liquid was redissolved in CH_2Cl_2 (10 mL) and washed with 5% aq. LiCl (3 × 10 mL). The organic layer was dried (hydrophobic frit) before concentration in vacuo to afford methyl (tert-butoxycarbonyl)homoserinate (**4c**) (575 mg, 2.47 mmol, 59% yield) as a yellow liquid (consistent with literature)[11]. ^{1}H NMR (400 MHz, $CDCl_3$) δ = 5.41 (br s, 1H), 4.45–4.56 (m, 1H), 3.78 (s, 3H), 3.61–3.75 (m, 2H), 3.13 (br s, 1H), 2.10–2.21 (m, 1H), 1.60–1.70 (m, 1H), 1.46 (s, 9H). ^{13}C NMR (101 MHz, $CDCl_3$) δ = 173.2, 156.3, 80.3, 58.3, 52.4, 50.7, 35.9, 28.2. LC-MS (CSH~2min_HpH) t_r = 0.70 min, [M+H$^+$–Boc] = 133.95, (92% purity).

methyl *N*-(tert-butoxycarbonyl)-*O*-(4-fluorophenyl)homoserinate (4d)

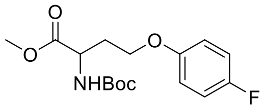

To a stirring solution of **4c** (402 mg, 1.21 mmol), triphenylphosphine (475 mg, 1.81 mmol) and 4-fluorophenol (135 mg, 1.21 mmol) in THF (8 mL) at 0 °C under nitrogen was added a solution of DIAD (0.35 mL, 1.81 mmol) in THF (2 mL), dropwise over 10 min. The resultant solution was stirred under nitrogen for a further 22.5 h, allowing the reaction to warm to rt. The reaction mixture was concentrated in vacuo to afford a yellow oil. The oil was stirred in TBME (4 mL) at 0 °C for 0.5 h. The solid that precipitated was filtered off. The filtrate was concentrated in vacuo to afford a yellow oil. The oil was dissolved in CH_2Cl_2 and purified by flash column chromatography (silica, 0–100% EtOAc in cyclohexane). The relevant fraction(s) were concentrated in vacuo to afford the desired product as a colourless oil. Some fractions overlapped with 4-fluorophenol were also concentrated in vacuo to afford a colourless oil. In this case, the crude oil was dissolved in CH_2Cl_2 (10 mL) and 2M aq. NaOH (10 mL) before shaking and separating. The organic layer was dried (hydrophobic frit) and concentrated in vacuo to afford the desired product as a colourless oil. The two samples were combined to afford methyl *N*-(tert-butoxycarbonyl)-*O*-(4-fluorophenyl)homoserinate (**4d**) (180 mg, 0.55 mmol, 46% yield) as a colourless oil. Infrared spectrum: ν_{max} (oil): 3360, 2978, 1710, 1505, 1202, 1159, 1049, 829 cm^{-1}. ^{1}H NMR (400 MHz, $CDCl_3$) δ = 6.86–7.05 (m, 2H), 6.73–6.86 (m, 2H), 5.27 (br s, 1H), 4.49 (br s, 1H), 4.00 (t, *J* = 5.9 Hz, 2H), 3.75 (s, 3H) 2.27– 2.38 (m, 1H), 2.13–2.25 (m, 1H), 1.43 (s, 9H). ^{13}C NMR (101 MHz, $CDCl_3$) δ = 172.7, 157.4 (d, $^{1}J_{C–F}$ = 239.1 Hz), 155.3, 154.6, 115.8 (d, $^{2}J_{C–F}$ = 22.7 Hz), 115.6 (d, $^{3}J_{C–F}$ = 8.1 Hz), 80.1, 64.8, 52.4, 51.2, 31.9, 28.3. ^{19}F NMR {^{1}H} (376 MHz,$CDCl_3$) δ = –123.6. LC-MS (CSH~2min_HpH) t_r = 1.20 min, $[M+H]^+$ = 326.13, (94% purity). HRMS: ($C_{16}H_{22}NO_5F$) $[M+H^+]$ requires 328.1482, found $[M+H^+–{}^{t}Boc]$ 228.1043.

methyl *O*-(4-fluorophenyl)-*N*-(2-phenyl-2*H*-tetrazole-5-carbonyl)homoserinate (4e)

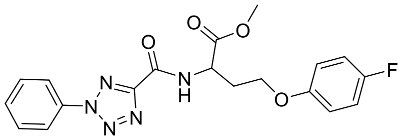

A solution of **4d** (140 mg, 0.43 mmol) and TFA (0.30 mL, 3.85 mmol) in CH_2Cl_2 (3 mL) was stirred at rt for 2.25 h. The reaction mixture was concentrated in vacuo to afford a colourless oil. A separate solution of **S1** (80 mg, 0.42 mmol), HATU (192 mg, 0.51 mmol) and DIPEA (0.22 mL, 1.26 mmol) in DMF (1 mL) was stirred at rt for 0.25 h. At which point a solution of the colourless oil (96 mg, 0.42 mmol) in DMF (1 mL) was added and the resultant solution was stirred at rt for a further 1 h. The reaction mixture was purified by MDAP (HPH_MethC_Ext). The relevant fractions were concentrated in vacuo to afford methyl *O*-(4-fluorophenyl)-*N*-(2-phenyl-2*H*-tetrazole-5-carbonyl)homoserinate (**4e**) (20 mg, 0.05 mmol, 12% yield) as a yellow solid. Infrared spectrum: ν_{max} (solid): 3393, 1750, 1693, 1546, 1507, 1198, 828, 769 cm^{-1}. ^{1}H NMR (400 MHz, $CDCl_3$) δ = 8.18–8.22 (m, 2H), 8.15 (br d, *J* = 7.3 Hz, 1H), 7.51–7.62 (m, 3H), 6.94–7.01 (m, 2H), 6.85–6.92 (m, 2H), 5.09 (ddd, *J* = 7.3, 5.9, 4.9 Hz, 1H), 4.05–4.16 (m, 2H), 3.81 (s, 3H), 2.50–2.59 (m, 1H), 2.40–2.50 (m, 1H). ^{13}C NMR (151 MHz, $CDCl_3$) δ = 171.3, 159.4, 157.6 (d, $^{1}J_{C–F}$ = 238.8 Hz), 156.3, 154.3 (d, $^{4}J_{C-F}$ = 2.2 Hz), 136.4, 130.6, 129.8, 120.3, 115.9 (d, $^{2}J_{C–F}$ = 23.2 Hz), 115.6 (d, $^{3}J_{C–F}$ = 8.3 Hz), 65.0, 52.8, 50.8, 31.2. ^{19}F {^{1}H} NMR (376 MHz,$CDCl_3$) δ = –123.2. LC-MS (CSH~2min_HpH) t_r = 1.21 min, $[M+H]^+$ = 400.09, (100% purity). HRMS: ($C_{19}H_{18}N_5O_4F$) $[M+H^+]$ requires 400.1343, found $[M+H^+]$ 400.1424.

O-(4-fluorophenyl)-N-(2-phenyl-2H-tetrazole-5-carbonyl)homoserine (4a)

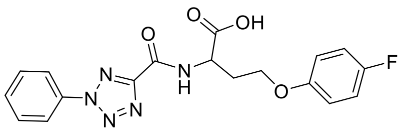

A solution of **4e** (12 mg, 0.03 mmol) and lithium hydroxide (0.94 mg, 0.04 mmol) in 3:1 THF:water (1.33 mL) was stirred at rt for 0.25 h. The reaction mixture was concentrated in vacuo to afford a yellow solid. The solid was suspended in water (2 mL) and 2M aq. HCl was added (2 mL). The product was extracted with EtOAc (4 × 4 mL). The combined organic layers were dried (hydrophobic frit) before being concentrated in vacuo to afford *O*-(4-fluorophenyl)-*N*-(2-phenyl-2*H*-tetrazole-5-carbonyl)homoserine (**4a**) (11 mg, 0.03 mmol, 95% yield) as a yellow oil. Infrared spectrum: ν_{max} (oil): 2923, 1688, 1550, 1505, 1201, 1006, 827, 759, 681 cm^{-1}. ^{1}H NMR (400 MHz, DMSO-d_6) δ = 12.92 (br s, 1H), 9.37 (d, *J* = 7.8 Hz, 1H), 8.11–8.17 (m, 2H), 7.62–7.74 (m, 3H), 7.05–7.13 (m, 2H), 6.92–6.98 (m, 2H), 4.71 (ddd, *J* = 9.3, 7.8, 4.9 Hz, 1H), 3.99–4.14 (m, 2H), 2.26–2.42 (m, 2H). ^{13}C NMR (151 MHz, DMSO-d_6) δ = 172.4, 159.6, 156.4, 156.5 (d, $^{1}J_{C–F}$ = 236.1 Hz), 154.7 (d, $^{4}J_{C–F}$ = 1.7 Hz), 136.0, 130.8, 130.2, 120.4, 115.8 (d, $^{3}J_{C–F}$ = 5.5 Hz), 115.7 (d, $^{2}J_{C–F}$ = 10.0 Hz), 64.9, 49.6, 29.9. ^{19}F {^{1}H} NMR (376 MHz, DMSO-d_6) δ = –123.9. LC-MS (CSH~2min_HpH) t_r = 0.79 min, [M+H$^+$] = 386.05, (99% purity). HRMS: ($C_{18}H_{16}N_5O_4F$) [M+H]$^+$ requires 386.1186, found [M+H$^+$] 386.1268.

8. References

1. K. Teegardin, J. I. Day, J. Chan and J. Weaver, *Org. Process Res. Dev.*, 2016, **20**, 1156-1163.
2. C. K. Prier, D. A. Rankic and D. W. C. MacMillan, *Chem. Rev.*, 2013, **113**, 5322-5363.
3. S. Yakubov and J. P. Barham, *Beilstein J. Org. Chem.*, 2020, **16**, 2151-2192.
4. N. A. Romero and D. A. Nicewicz, *Chem. Rev.*, 2016, **116**, 10075-10166.
5. F. Strieth-Kalthoff, M. J. James, M. Teders, L. Pitzer and F. Glorius, *Chem. Soc. Rev.*, 2018, **47**, 7190-7202.
6. H. E. Bonfield, K. Mercer, A. Diaz-Rodriguez, G. C. Cook, B. S. J. McKay, P. Slade, G. M. Taylor, W. X. Ooi, J. D. Williams, J. P. M. Roberts, J. A. Murphy, L. Schmermund, W. Kroutil, T. Mielke, J. Cartwright, G. Grogan and L. J. Edwards, *ChemPhotoChem*, 2020, **4**, 45-51.
7. R. P. Thomas, R. E. Heap, F. Zappacosta, E. K. Grant, P. Pogány, S. Besley, D. J. Fallon, M. M. Hann, D. House, N. C. O. Tomkinson and J. T. Bush, *Chem. Sci.*, 2021, **12**, 12098-12106.
8. A. J. Leo and D. Hoekman, *Perspect. Drug Discov. Des.*, 2000, **18**, 19-38.
9. J. Szegezdi and F. Csizmadia, *233rd ACS National Meeting*, 2007, https://chemaxon.com/poster/a-method-for-calculating-the-pka-values-of-small-and-large-molecules.
10. D. Kessler, M. Gmachl, A. Mantoulidis, L. J. Martin, A. Zoephel, M. Mayer, A. Gollner, D. Covini, S. Fischer, T. Gerstberger, T. Gmaschitz, C. Goodwin, P. Greb, D. Häring, W. Hela, J. Hoffmann, J. Karolyi-Oezguer, P. Knesl, S. Kornigg, M. Koegl, R. Kousek, L. Lamarre, F. Moser, S. Munico-Martinez, C. Peinsipp, J. Phan, J. Rinnenthal, J. Sai, C. Salamon, Y. Scherbantin, K. Schipany, R. Schnitzer, A. Schrenk, B. Sharps, G. Siszler, Q. Sun, A. Waterson, B. Wolkerstorfer, M. Zeeb, M. Pearson, S. W. Fesik and D. B. McConnell, *Proc. Natl. Acad. Sci. U.S.A*, 2019, **116**, 15823-15829.
11. *Pat. WO 03/018565*, 2003.
